# Comprehensive Profiling of Gene Expression in the Cerebral Cortex and Striatum of BTBRTF/ArtRbrc Mice Compared to C57BL/6J Mice

**DOI:** 10.3389/fncel.2020.595607

**Published:** 2020-12-10

**Authors:** Shota Mizuno, Jun-na Hirota, Chiaki Ishii, Hirohide Iwasaki, Yoshitake Sano, Teiichi Furuichi

**Affiliations:** ^1^Department of Applied Biological Science, Faculty of Science and Technology, Tokyo University of Science, Noda, Japan; ^2^Department of Anatomy, Gunma University Graduate School of Medicine, Maebashi, Japan

**Keywords:** *in situ* hybridization, co-expression network, bioinformatics analysis, transcriptome, BTBR mouse model, autism spectrum disorder, RNA-RNA interaction network

## Abstract

Mouse line BTBR *T*+ *Iptr3*^*tf*^/J (hereafter referred as to BTBR/J) is a mouse strain that shows lower sociability compared to the C57BL/6J mouse strain (B6) and thus is often utilized as a model for autism spectrum disorder (ASD). In this study, we utilized another subline, BTBRTF/ArtRbrc (hereafter referred as to BTBR/R), and analyzed the associated brain transcriptome compared to B6 mice using microarray analysis, quantitative RT-PCR analysis, various bioinformatics analyses, and *in situ* hybridization. We focused on the cerebral cortex and the striatum, both of which are thought to be brain circuits associated with ASD symptoms. The transcriptome profiling identified 1,280 differentially expressed genes (DEGs; 974 downregulated and 306 upregulated genes, including 498 non-coding RNAs [ncRNAs]) in BTBR/R mice compared to B6 mice. Among these DEGs, 53 genes were consistent with ASD-related genes already established. Gene Ontology (GO) enrichment analysis highlighted 78 annotations (GO terms) including DNA/chromatin regulation, transcriptional/translational regulation, intercellular signaling, metabolism, immune signaling, and neurotransmitter/synaptic transmission-related terms. RNA interaction analysis revealed novel RNA–RNA networks, including 227 ASD-related genes. Weighted correlation network analysis highlighted 10 enriched modules including DNA/chromatin regulation, neurotransmitter/synaptic transmission, and transcriptional/translational regulation. Finally, the behavioral analyses showed that, compared to B6 mice, BTBR/R mice have mild but significant deficits in social novelty recognition and repetitive behavior. In addition, the BTBR/R data were comprehensively compared with those reported in the previous studies of human subjects with ASD as well as ASD animal models, including BTBR/J mice. Our results allow us to propose potentially important genes, ncRNAs, and RNA interactions. Analysis of the altered brain transcriptome data of the BTBR/R and BTBR/J sublines can contribute to the understanding of the genetic underpinnings of autism susceptibility.

## Introduction

Autism spectrum disorder (ASD) is a complex neurodevelopmental disorder characterized by altered functionality across two symptom domains: (1) social and communication deficits, and (2) stereotyped repetitive behaviors with restricted interests (American Psychiatric Association, 2013). ASD is highly heterogeneous in terms of clinical symptoms and etiology. The current prevalence rate of ASD is ~1 in 40 children (Xu et al., 2018). As there is no cure for ASD, understanding its pathophysiology is an important health issue. ASD is highly heritable and is also affected by environmental factors. Recent advances in next generation sequencing have revealed more than 1,000 mutations associated with ASD (Devlin and Scherer, 2012). However, many of the genomic alterations identified thus far are rare variants that represent only a small fraction of cases of ASD (Chaste and Leboyer, 2012; Devlin and Scherer, 2012). Recent ASD studies performed at the molecular, cellular, and behavioral levels, including mouse model studies, suggest that ASD is clinically heterogeneous with diverse pathophysiological processes that lead to similar behavioral manifestations (de la Torre-Ubieta et al., 2016). Integrative analysis of large-scale genetic data reveals distinct gene networks affected in ASD, mainly related to synaptic function and formation within the brain. Network-based analysis of large-scale transcriptome data also highlights that co-expression modules related to synaptic development, neuronal activity, and immune function are deregulated in ASD (Voineagu et al., 2011; Gupta et al., 2014; de la Torre-Ubieta et al., 2016). Thus, the pathophysiological processes in ASD seem to converge on specific molecular pathways and networks, with a clear interplay between immune and synaptic functions (Estes and McAllister, 2015).

The black and tan brachyury (BTBR) mouse strain was originally created by Leslie Clarence Dunn (Columbia University) using stock obtained from Nadine Dobrovolskaia-Zavadskaia (Pasteur Institute); they were maintained by Karen Artzt (the University of Texas, Austin; named as “BTBRTF/Art” in Wu et al., 2007) after passing them to some researchers, according to Clee et al. (2005) and the Jackson Laboratory (Bar Harbor, ME). BTBRTF/Art was then distributed to and bred in many other laboratories, including the Jackson Laboratory (BTBR *T*+ *Iptr3*^*tf*^/J, MGI ID: 4452239, hereafter referred to as “BTBR/J”) from 1994 and the RIKEN BioResource Research Center (RBRC) (Tsukuba, Japan; BTBRTF/ArtRbrc, RBRC ID: 01206, hereafter referred to as “BTBR/R”) from 1987. Both BTBR subline BTBR/J and BTBR/R mice have the spontaneous mutations *T* (brachyury), *Itpr3*^*tf*^ (inositol 1,4,5-trisphosphate receptor 3, tufted), and *a*^*t*^ (black and tan). Interestingly, BTBR/J mice have been shown to have reduced social behavior relevant to ASD (Bolivar et al., 2007; Moy et al., 2007, 2008; McFarlane et al., 2008; Pobbe et al., 2010; Defensor et al., 2011; Pearson et al., 2011; Scattoni et al., 2011, 2013; Wöhr et al., 2011; Silverman et al., 2012, 2015). Gene transcriptional profiling in ASD has been performed by utilizing post-mortem brain samples. The restricted availability of brain tissue from humans with ASD represents a significant challenge. From this point of view, BTBR/J is a valuable animal model of ASD to analyze the molecular and pathological mechanism at the gene and protein expression level. For this purpose, BTBR/J mice have been subjected to transcriptome analyses of the hippocampus (Daimon et al., 2015; Provenzano et al., 2016; Gasparini et al., 2020), frontal cortex (Kratsman et al., 2016), dorsal striatum (Oron et al., 2019), and cerebellum (Shpyleva et al., 2014), as well as proteome analyses of cortical (Jasien et al., 2014; Wei et al., 2016) and hippocampal tissues (Jasien et al., 2014; Daimon et al., 2015).

In this study, we analyzed the brain transcriptome of BTBR/R mice that have enhanced turnover of dendritic spines in the anterior frontal cortex, similar to that seen in ASD model mice, namely patDp/+ with paternal duplication of chromosome 7c and *NLGN-3* R451C with a point mutation of human neuroligin-3, during the postnatal developmental stage (Isshiki et al., 2014). We performed a comprehensive brain transcriptome analysis between BTBR/R mice and C57BL/6J (hereinafter referred to as B6) mice with high sociality, using genome-wide microarray analysis, quantitative RT-PCR (qRT-PCR) analysis, various bioinformatics analyses, and *in situ* hybridization (ISH). We paid particular attention to the cerebral cortex and striatum, since altered cortico-striatal connectivity has been suggested to be present in patients with ASD (Abbott et al., 2018) and in an ASD model mouse with complete knockout (KO) of *Shank3* (Wang et al., 2016). Our results revealed the differential transcription profiles in microarray expression levels as well as cellular-regional levels in the cerebral cortex and striatum of BTBR/R mice and B6 mice. Some genes and non-coding RNAs (ncRNAs) that we detected have also been reported or suggested to be ASD-related genes, or as differentially expressed genes (DEGs) in ASD animal models including BTBR/J mice. Moreover, our behavioral analysis data suggested that BTBR/R mice have a slight autistic-like tendency in terms of social novelty recognition and stereotypic behavior. Taken together, the results of our study suggest the genetic aspects of BTBR/R mice brain function, which is informative to the understanding of the genetics of the observed behavioral defects.

## Materials and Methods

### Animals

All experimental protocols were evaluated and approved by the Regulation for Animal Research at Tokyo University of Science. All experiments were conducted in accordance with the Regulations for Animal Research at The University of Tokyo and Tokyo University of Science. The BTBR/R mouse strain (BTBRTF/ArtRbrc, RBRC01206) was provided by the RBRC through the National Bio-Resource Project of the Ministry of Education, Culture, Sports, Science and Technology, Japan (Isshiki et al., 2014) and were housed in the animal facility at The University of Tokyo and Tokyo University of Science on 12-h light and dark cycle from 8 a.m. to 8 p.m. The B6 mouse strain (C57BL/6J) was purchased from the Japan SLC, Inc. (Hamamatsu, Japan) and were housed in the animal facility at Tokyo University of Science on 12-h light and dark cycle from 8 a.m. to 8 p.m.

### Microarray Analysis

Male BTBR/R (4–6 months of age) and B6 (4 months of age) mice were deeply anesthetized with Somnopentyl (64.8 mg/ml pentobarbital sodium, 150 mg/kg, i.p.; Kyoritsu Seiyaku Corporation, Tokyo, Japan) and were decapitated. Brain tissue samples (cerebral cortex and striatum) from four mice (*N* = 4) of each strain were dissected out and treated in an RNA-stabilizing agent (RNAlater, Qiagen, Hilden, Germany). Microarray analysis was performed by RIKEN Genesis Co., Ltd. (Tokyo, Japan). Briefly, total RNA was extracted with the RNeasy Plus Universal Mini Kit (Qiagen) and RNA quality was analyzed using a 2100 Bioanalyzer (Agilent, Santa Clara, CA, USA) and NanoDrop (Thermo Fisher Scientific, Waltham, MA, USA). Only RNA with a high (>8) RNA integrity number was selected. Cys3-labeled complementary RNAs (cRNAs) were prepared using the Low Input Quick Amp Labeling Kit, One-Color (Agilent), and were analyzed by using the SurePrint G3 Mouse Gene Expression 8x60K v2 microarray (Agilent) that features complete coverage of established RefSeq coding transcripts (NM sequences) from the latest build and updated long ncRNA (lncRNA) content (27,122 Entrez genes and 4,578 lncRNAs), according to the manufacturer's protocol. Hybridization images were acquired using the DNA Microarray Scanner (Agilent) and were analyzed with Feature Extraction Software version 10.7.3.1 (Agilent). Signal analysis and batch normalization were carried out using GeneSpring GX (Agilent). The gene expression datasets of the BTBR/R and B6 mice have been deposited in the NCBI Gene Expression Omnibus database (accession number GSE156646).

### Analysis of DEGs

Gene expression in BTBR/R mice was normalized to that of B6 mice to obtain the fold change (FC) of all probes (the value obtained in B6 was set to 1). The probes were tested for differential expression using unpaired two-tailed Student's *t*-tests, and the false discovery rate (FDR) was calculated using *P*-values (Benjamini and Hochberg, 1995). An FDR cut-off of <0.05 and FC of ≥ absolute 2.0 were applied. The DEGs are listed in Supplementary Table 1.

### Bioinformatics Analyses

Transcript types (such as splicing variants) of the detected genes were defined using the gene databases GenBank (NCBI; Sayers et al., 2020a), Refseq (NCBI; O'Leary et al., 2016), Ensembl release 100 (EMBI-EBI; Yates et al., 2020) and Agilent microarray's information. Each transcript type was categorized into “Protein coding,” “Non-coding RNA,” “Pseudogene,” and “Other.” The category descriptions are listed in Supplementary Table 2.

#### Enrichment Analysis of GO Annotations and KEGG Pathways by DAVID

For gene description and pathway enrichment analyses of the DEGs, we utilized the Gene Ontology (GO) and Kyoto Encyclopedia of Genes and Genomes (KEGG) bioinformatics databases and visualized as well as integrated the DEGs based on these resources using the Database for Annotation, Visualization, and Integrated Discovery (DAVID) Functional Annotation tool (Huang et al., 2009a,b; Leidos Biomedical Research). For all analyses, the background of animal species was set to *Mus musculus*, and the enrichment threshold was a DAVID-corrected *P* < 0.05. The data of enriched GO descriptions and KEGG pathways are listed in Supplementary Tables 3, 4.

### Weight Correlation Network Analysis (WGCNA)

WGCNA was run with R version 4.0.2 and the WGCNA package version 1.69 (Langfelder and Horvath, 2008), using microarray data of the most informative (top 20%) probes in terms of per-probe variance, in accordance with previous work (Provenzano et al., 2016). We used the similarity between gene expression profiles to construct a similarity matrix based on pairwise Pearson correlation coefficient matrices. The similarity matrix was transformed into an adjacency matrix using a power adjacency function (Zhang and Horvath, 2005). In this study, we chose the power of 14 that this value was calculated by using the integration function (pickSoftThreshold) in the WGCNA software package. We then calculated the topological overlap measure (TOM), which is a robust measure of network interconnectedness, using an adjacency matrix. Finally, we performed an average linkage hierarchical clustering according to the TOM-based dissimilarity measure. The tree-cut algorithm method was adopted to identify the module of gene co-expression with a minModuleSize of 30 and a mergeCutHeight of 0.25. GO enrichment analysis for each module was performed using the integration function (GOenrichmentAnalysis) of the WGCNA software package. The module genes and the enriched GO results are listed in Supplementary Tables 5, 6.

#### ENCORI and Cytoscape Analyses

RNA interaction data were obtained using the RNA target database ENCORI (Li et al., 2014). In this analysis, we used each DEG and their targets as network nodes. The interactions network was analyzed and visualized by the network visualization software Cytoscape version 3.8.0 (https://cytoscape.org/). The RNA interactions are listed in Supplementary Table 7.

#### AutDB Search

Genes associated with ASD referenced the autism database AutDB (MindSpec; Pereanu et al., 2018). “Gene Score” and “Syndromic” annotations were obtained from the autism gene database SFARI (https://gene.sfari.org/). Mouse ASD gene orthologs were detected using the gene databases Homologene (Sayers et al., 2020b), Ensembl release 100 (Yates et al., 2020) and Mouse Genome Database (The Jackson Laboratory; Bult et al., 2019). The list of Mouse ASD gene orthologs and DEGs identical to ASD-related genes registered in the AutDB are shown in Supplementary Tables 8, 9.

### qRT-PCR

cDNA libraries were prepared from total corticostriatal RNAs using the SuperScript IV VILO Master Mix (Thermo Fisher Scientific) according to the manufacturer's protocol. qRT-PCR was performed using 7300 systems (Thermo Fisher Scientific) with real-time detection of fluorescence, using the Power SYBR Green PCR Master Mix (Thermo Fisher Scientific). Mouse glyceraldehyde-3-phosphate dehydrogenase was used as a standard for quantification. Primer sequences (Integrated DNA Technologies, Coralville, IA, USA) are listed in Supplementary Table 10. Expression analyses were performed using the 7300 system SDS software version 1.4 (Thermo Fisher Scientific). Quantitative values were obtained from the threshold cycle (CT) number. Fold change was calculated using the ΔΔCT method, with B6 sample data as the control. All transcripts were run in duplicates and plotted as the average of two independent reactions obtained from each cDNA.

### ISH

ISH was performed as described previously, with small modification (Sano et al., 2014). Male BTBR/R (2–3 months of age) and B6 (2–4 months of age) were used for this experiment. Brains were perfused with 4% paraformaldehyde (PFA), harvested, post-fixed with 4% PFA at 4°C for 1 d, and then equilibrated in 30% (w/v) sucrose in phosphate-buffered saline (PBS). Coronal sections (50 μm thickness) were prepared using a cryostat. All steps were performed at room temperature unless indicated otherwise. Sections were incubated with methanol (MeOH) for 2 h, then washed three times for 10 min in PBS containing 0.1% Tween-20 (PBST), incubated with 3.3 μg/ml proteinase K (Sigma-Aldrich, St. Louis, MO, USA) in ProK buffer (0.1 M Tris HCl, pH 8.0, 50 mM EDTA) for 30 min at 37°C, incubated with 0.25% Acetic anhydride in 0.1 M triethanolamine, pH 7.0 for 10 min, washed twice for 5 min in PBST and, finally, incubated with hybridization buffer (5 × SSC, 50% formamide, 0.1% Tween-20, 5x Denhardt's solution) for 1 h at 60°C. Prior to hybridization, digoxigenin (DIG)-labeled cRNA probes in hybridization buffer were denatured at 80°C for 5 min and then quickly cooled on ice for 10 min. cRNA probes were generated using a DIG RNA labeling kit (Roche, Penzberg, Germany). Hybridization was performed at 60°C overnight. Sections were washed in 2 × SSC containing 50% formamide and 0.1% Tween-20 (SSCT) twice for 20 min, incubated with 20 μg/ml RNase (Nippon Gene, Tokyo, Japan) in RNase buffer (10 mM Tris-HCl, pH 8.0, 1 mM EDTA, 0.5 M NaCl) for 30 min at 37°C, washed in 2 × SSCT twice for 15 min at 37°C, and 0.2 × SSCT twice for 15 min at 37°C. Then, the sections were incubated with 1% blocking reagent (Roche; 10 mM maleic acid, 15 mM NaCl, pH 7.5) for 1 h, and finally incubated with alkaline phosphatase-conjugated anti-DIG antibody (1:2,000, Roche) in blocking reagent at 4°C overnight. The sections were washed three times in TNT (0.1 M Tris-HCl, pH 7.5, 0.15 M NaCl, 0.05% Tween-20) for 15 min. For staining with nitroblue tetrazolium chloride/5-bromo-4-chloro-3-indolyl phosphate 4-toluidine salt (NBT/BCIP), the signal was developed in 2% (v/v) NBT/BCIP stock solution (Roche) diluted in TS9.5 (0.1 M NaCl, 10 mM MgCl_2_, 0.1 M Tris pH 9.5) at room temperature overnight. Sections were imaged using a NanoZoomer Digital Pathology virtual slide scanner (Hamamatsu Photonics, Hamamatsu, Japan). ISH images were verified by analyzing at least three different brain sections from 1–3 mice for each strain. ISH data with clarity as well as reproducibility in terms of signal intensities and patterns were used in this study, although many other genes were also analyzed. The probe sequences are listed in Supplementary Table 11.

### Statistical Analysis

All statistical analyses were performed in Excel 2019 (Microsoft, Redmond, WA, USA). Datasets were analyzed for significance using unpaired two-tailed Student's *t*-tests. All data are presented as mean ± SEM. In this study, *P* < 0.05 were considered significant. The additional information on each statistical analysis is described in the corresponding sections of the Materials and Methods section and the figure legends.

## Results

### Differential Cortical and Striatal Gene Expression Between BTBR/R and B6 Mice

**Figure 1:**
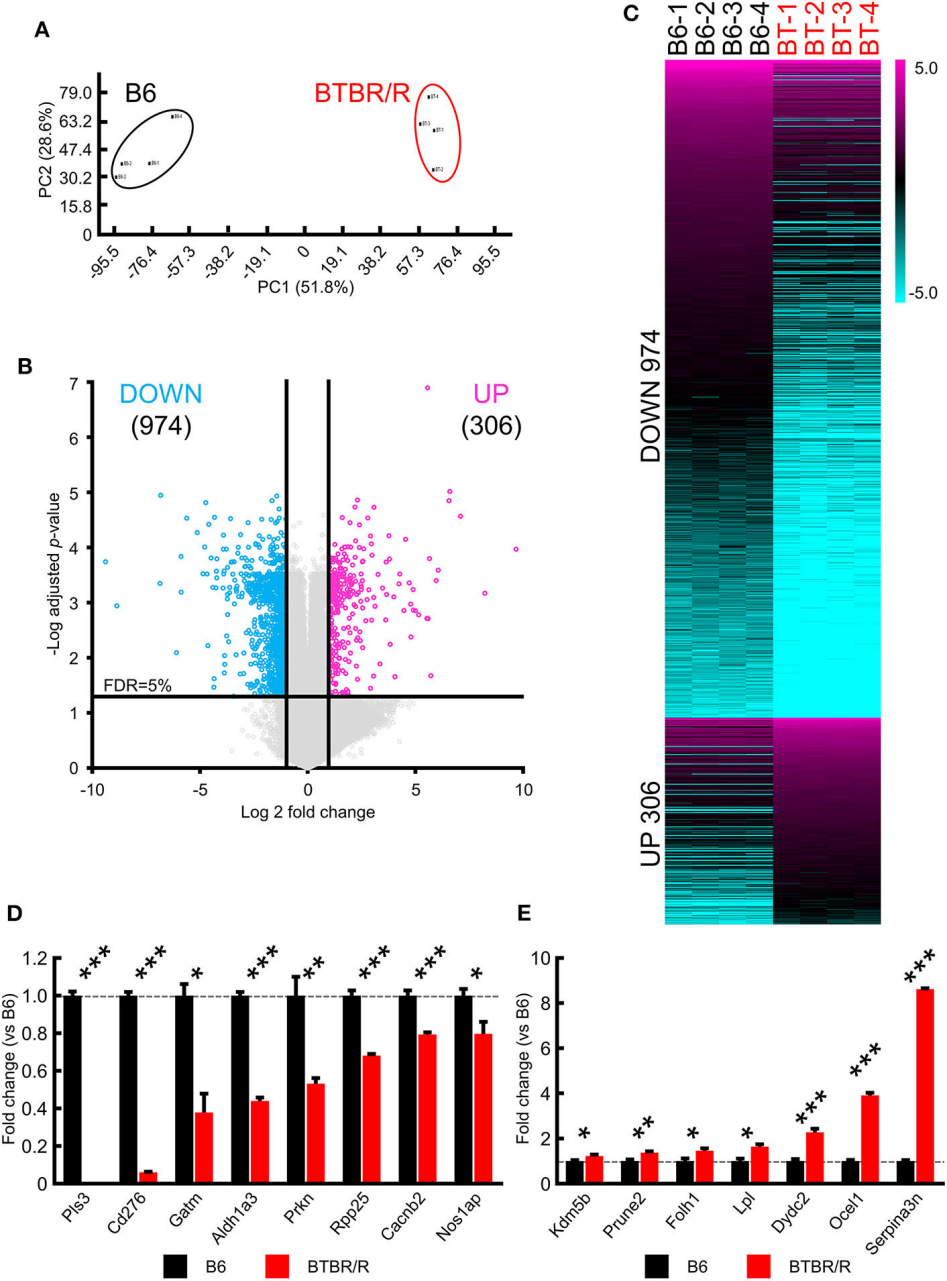
Significant alterations in BTBR/R cortical and striatal gene expression compared to B6. Gene expression changes in the cortical and striatal tissues from BTBR/R mice compared to tissues from B6 mice controls. **(A)** Principal component analysis of gene expression profiles of all samples. **(B)** Volcano plots depicting fold change vs. adjusted *P*-values for gene expression between BTBR/R and B6 mice. **(C)** Heat map of all differentially expressed genes between BTBR/R mice and B6 mice. The threshold was set to fold change ≥2 and *t*-test adjusted *P*-value ≤ 0.05 as illustrated in B. qRT-PCR-mediated validation revealed significantly downregulated **(D)** and upregulated **(E)** transcripts. Histogram black bars represent B6 mice data and red bars represent BTBR/R mice data. Unpaired two-tailed Student's *t*-test; *P < 0.05,**P < 0.01,***P < 0.001.

To compare the transcriptome features in the cerebral cortex and striatum between BTBR/R and B6 mice, we performed DNA microarray analysis for a total of 27,122 Entrez genes and 4,578 lncRNAs. Principal component analysis showed that the total RNA expression in BTBR/R and B6 mice were separated into two distinct groups (Figure 1A), indicating that each strain has distinct gene expression patterns and there is no outlier of the arrays. The data indicated that BTBR/R mice exhibited the differential expression of 1,280 transcripts (974 downregulated, 306 upregulated) in the cerebral cortex and striatum compared to B6 mice (Figures 1B,C, Supplementary Table 1).

For verification of the microarray data, we performed qRT-PCR analyses in the cerebral cortex and striatum samples between BTBR/R and B6 mice for 15 DEGs: eight downregulated genes for plastin 3 (*Pls3*), CD276 antigen (*Cd276*), glycine amidinotransferase (*Gatm*), aldehyde dehydrogenase family 1, subfamily A3 (*Aldh1a3*), parkin RBR E3 ubiquitin protein ligase (*Prkn*), ribonuclease P and MRP subunit p25 (*Rpp25*), calcium channel, voltage-dependent, beta 2 subunit (*Cacnb2*), and nitric oxide synthase 1 adaptor protein (*Nos1ap*; Figure 1D); and seven upregulated genes for prune homolog 2 (*Prune2*), folate hydrolase 1 (*Folh1*), lipoprotein lipase (*Lpl*), DPY30 domain containing 2 (*Dydc2*), lysine-specific demethylase 5B (*Kdm5b*), occludin/ELL domain containing 1 (*Ocel1*), and serine peptidase inhibitor, clade A, member 3N (*Serpina3n*; Figure 1E). Only ASD-related DEGs and DEGs with high fold change were selected. As a result, we confirmed that the increased and decreased expression of tested genes were consistent with the results of the microarray analysis.

**Figure 2:**
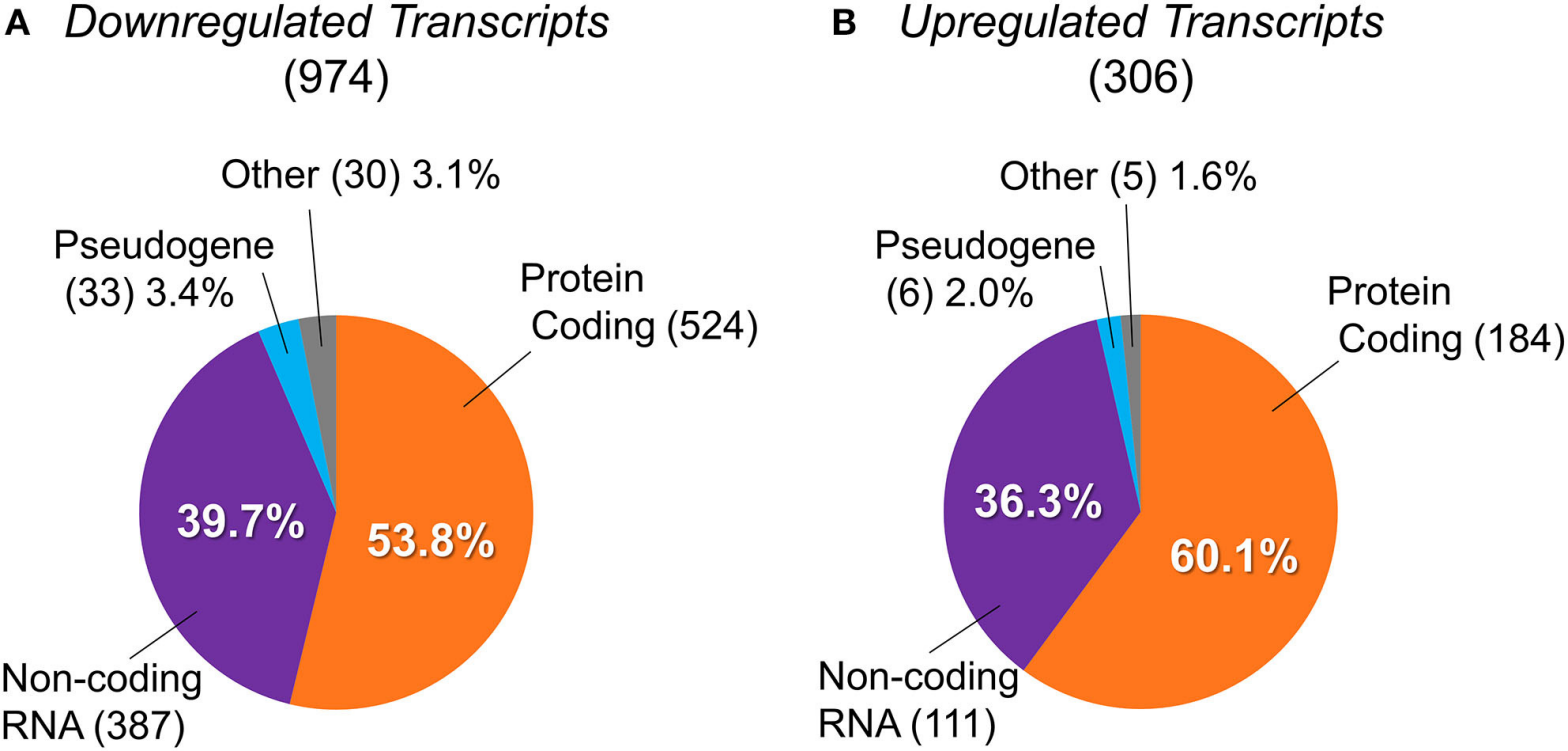
Classification of differentially expressed transcripts into protein-coding genes, non-coding RNAs, pseudogenes, and others. The transcript types of differentially expressed genes between BTBR/R mice and B6 mice. **(A)** 974 downregulated transcripts were classified into 524 protein-coding genes (53.8%), 387 non-coding RNAs (ncRNAs; 39.7%), 33 pseudogenes (3.4%), and 30 others (3.1%). **(B)** 306 upregulated transcripts were classified into 184 protein-coding genes (60.1%), 111 ncRNAs (36.3%), 6 pseudogenes (2.0%), and 5 others (1.6%).

Using the NCBI and Ensembl databases, we classified types of the differentially expressed transcripts identified, as shown in Figure 2. Downregulated and upregulated transcript groups consisted of 524 and 184 protein-coding transcripts, 387 and 111 non-coding transcripts, 33 and 6 pseudogenes, and 30 and 5 other transcripts, respectively (Supplementary Table 2).

#### ENCORI and Cytoscape RNA–RNA Interaction Networks Analysis

**Figure 3:**
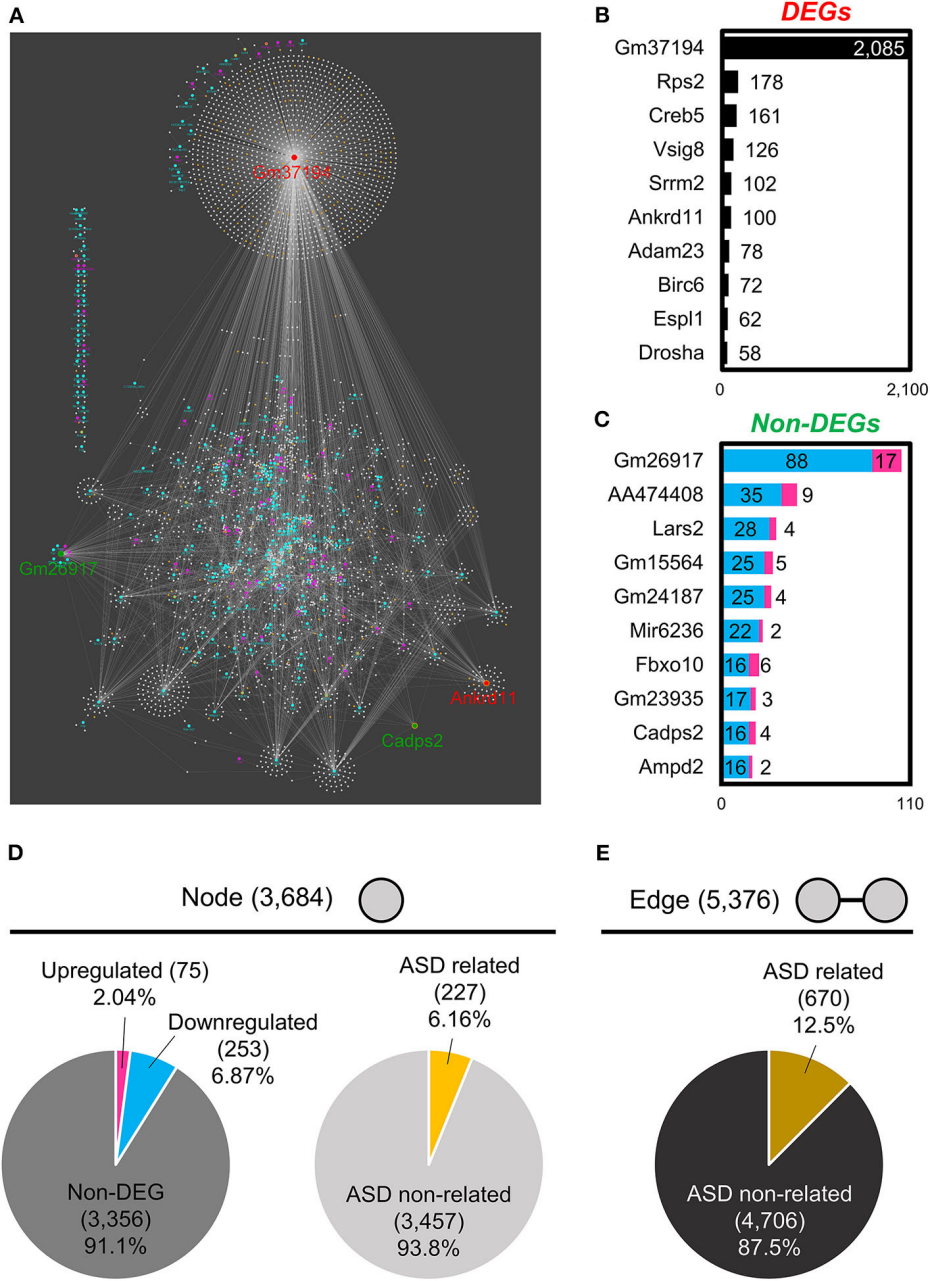
RNA–RNA interaction network analysis of BTBR/R gene expression. **(A)** RNA–RNA interaction networks constructed by 328 differentially expressed genes (DEGs) and 3,356 non-DEGs. The top 10 hub genes of DEGs **(B)** and non-DEGs **(C)** were based on the number of edges. Node's and Edge's attribute. **(D)** Nodes were classified into upregulated genes (2.04%), downregulated genes (6.87%) and non-DEGs (91.1%). Nodes were also classified into autism spectrum disorder (ASD)-related genes (6.16%) and ASD non-related genes (93.8%). **(E)** Edges constructed by ASD-related genes were 12.5%.

To understand the coordinated expression of RNAs, we focused on RNA–RNA interactions. By using the Cytoscape network analysis tool, we analyzed the relationship between specific RNA and its binding target RNAs. For DEGs, both the “DEG and DEG” interactions and the “DEG and non-DEG” interactions were analyzed, while for non-DEGs only the “non-DEG and DEG” interactions were analyzed. We found that DEGs had an RNA interaction network with 3,684 “nodes” (genes) and 5,376 “edges” (interactions; Figure 3A, Supplementary Table 7). These network nodes included 75 upregulated genes and 253 downregulated genes (Figure 3D). Moreover, this network had 227 ASD-related genes connected with 670 edges (Figures 3D,E). Figures 3B,C list the top 10 “hub” genes that interact highly with DEG and non-DEG nodes, in terms of the number of nodes (Figures 3B,C).

**Figure 4:**
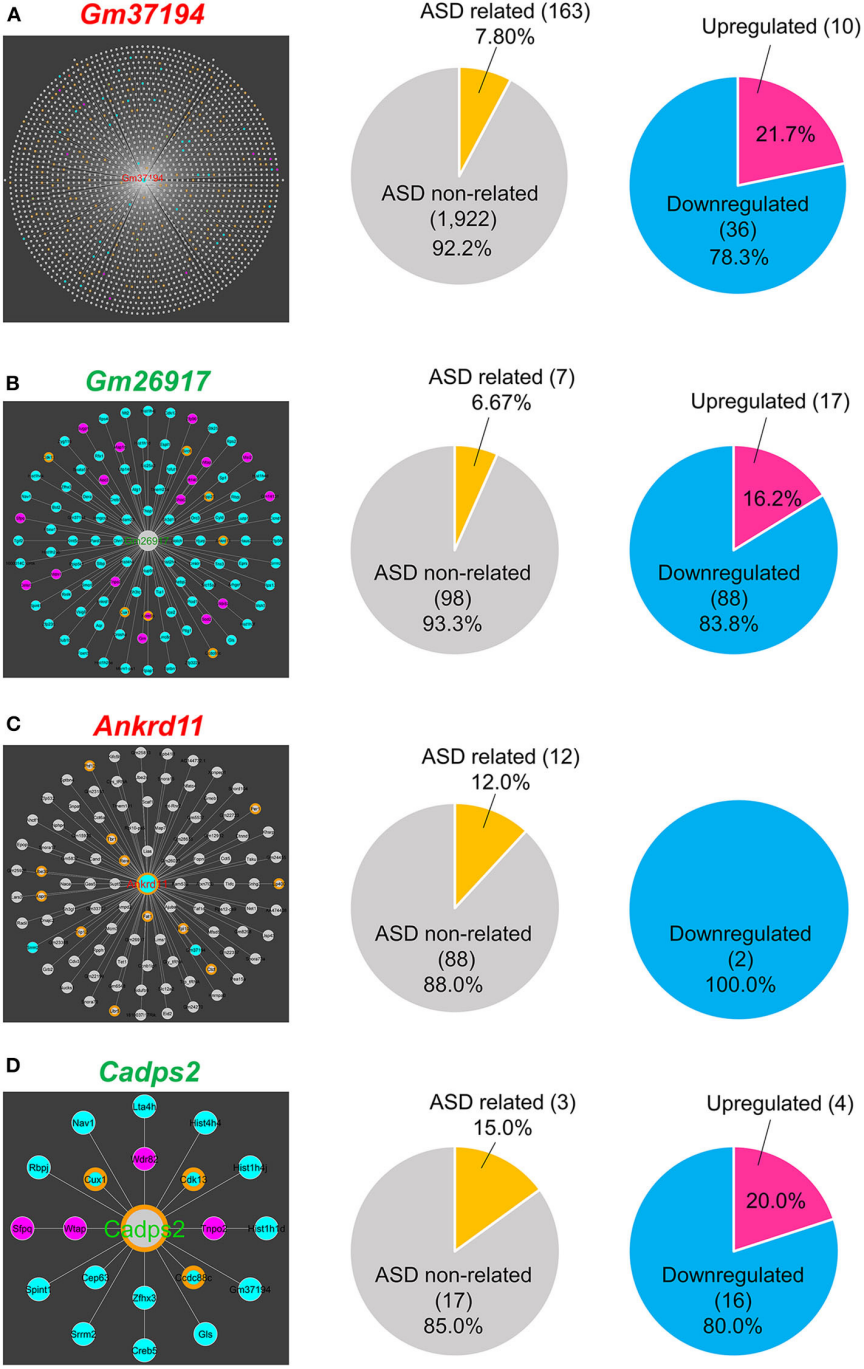
RNA-RNA interaction network hubs. **(A)** *Gm37194* interaction network. *Gm37194*'s nodes had 163 ASD-related genes (7.80%) and 46 DEGs (2.20%). **(B)** *Gm26917* interaction network. *Gm26917*'s nodes had seven ASD-related genes (6.67%), 17 upregulated genes (16.2%), and 88 downregulated genes (83.8%). **(C)** *Ankrd11* interaction network. *Ankrd11*'s nodes had 12 ASD-related genes (12.0%) and 2 DEGs (2.0%). **(D)** *Cadps2* interaction network. *Cadps2*'s nodes had three ASD-related genes (15.0%), four upregulated genes (20.0%), and 16 downregulated genes (80.0%).

In DEGs, predicted gene 37194 (*Gm37194*; downregulated) was the node with the largest RNA–RNA interaction network and acted as a hub RNA interacting with 2,085 target RNAs, including 163 ASD-related genes (Figure 4A). In non-DEGs, predicted gene 26917 (*Gm26917*; non-DEG) was the node with the largest RNA–RNA interaction network and acted as a hub interacting with 105 nodes including seven ASD-related genes (Figure 4B). In ASD-related genes, ankyrin repeat domain 11 (*Ankrd11*; downregulated) and calcium dependent activator protein for secretion 2 (*Cadps2*; non-DEG) were nodes with the largest RNA–RNA interaction networks. *Ankrd11* was a node for 100 RNAs, only two of which were DEGs (both downregulated) and 12 of which were ASD-related genes (Figure 4C). *Cadps2* was not a DEG but was a node for 20 RNAs, all of which were DEGs (4 upregulated, 16 downregulated), including three ASD-related genes (*Ccdc88c, Cdk13*, and *Cux1*; Figure 4D).

Collectively, our comparative whole genome-wide microarray analysis between BTBR/R and B6 found 708 differentially expressed protein-coding genes (524 downregulated and 184 upregulated) that corresponds to ~3.2% of the total protein-coding genes in mice (22,519 in Ensembl as of April 2020), suggesting complicated alterations in transcriptional regulation of the cerebral cortex and striatum between the two mouse strains, which is also implied by the altered expression of 498 ncRNAs and 39 pseudogenes that may be associated with the differential regulation of transcription and translation between two strains via RNA–RNA interactions.

### Bioinformatic Characterization of Differentially Expressed Genes Between BTBR/R and B6 Mice

To functionally characterize the differential gene expression between BTBR/R and B6 mice, identified DEGs were further subjected to various bioinformatics analyses.

#### Gene Ontology (GO) Functional Classification

**Figure 5:**
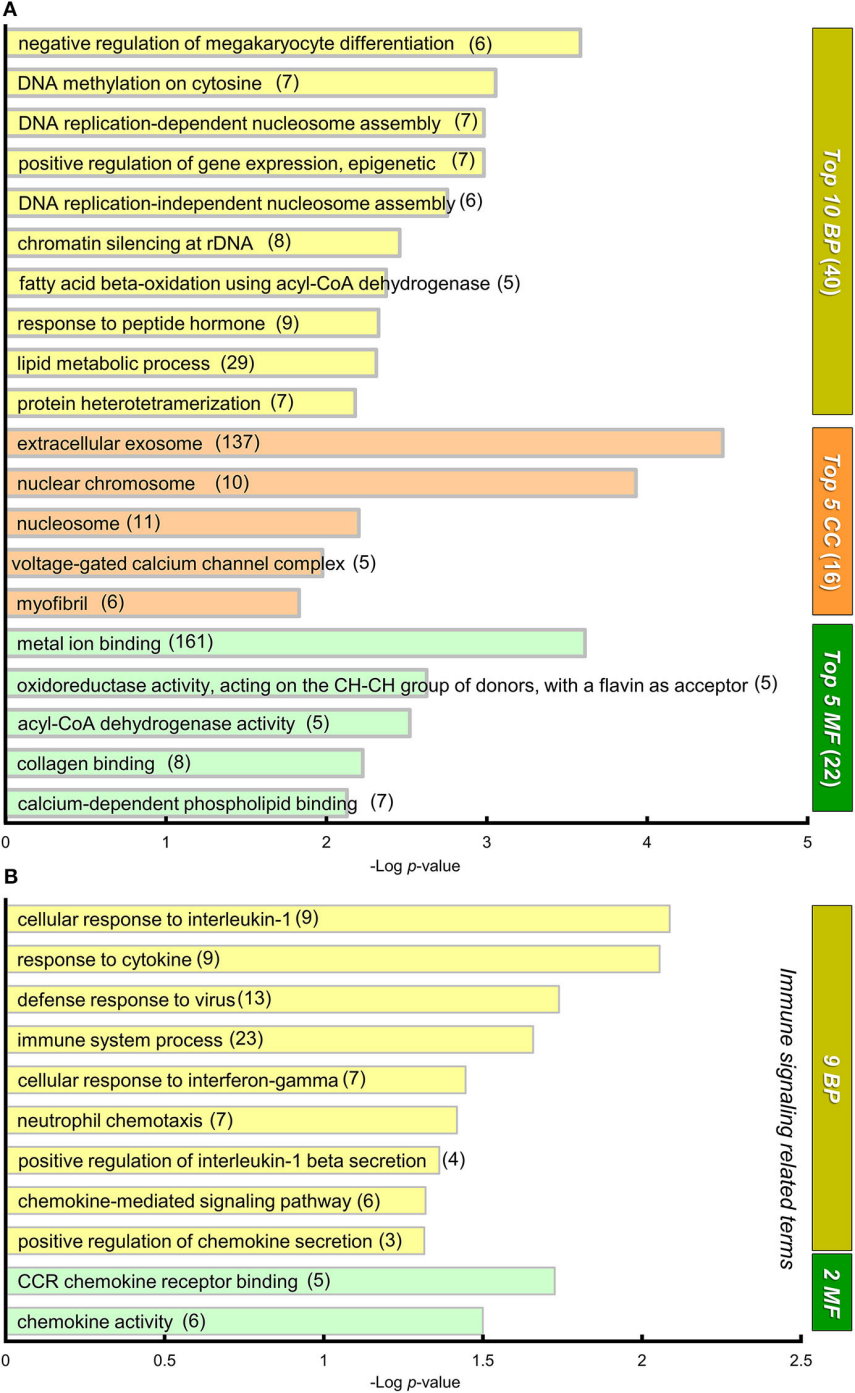
GO annotation and DAVID functional classification of DEGs. **(A)** A graph showing gene ontology (GO) annotations of differentially expressed genes (DEGs) with high statistical significance. DAVID functional classification. DEGs were analyzed for enrichment in GO using DAVID, with an adjusted *P* < 0.05. For each category, adjusted *P*-value is indicated by the length of the horizontal bars (Log *P*-value). DEGs were enriched in 78 GO annotations from three sub-ontologies: 40 biological processes (BP), 16 cellular components (CC), and 22 molecular functions (MF) with statistical significance (Supplementary Table 3). Among them, the top 10 BP, top 5 MF, and top 5 CC GO terms are shown in this graph. Numerals in parentheses indicate numbers of DEGs annotated to the terms. **(B)** A graph showing 9 and 2 “immune signaling related” GO terms from BP and MF, respectively. The DEGs were annotated to these 11 “immune signaling” -related GO terms that showed statistical significance.

To gain an insight into the biological significance in altered gene expression between BTBR/R and B6 mice, we performed the functional gene classification of DEGs by the GO enrichment analysis using the functional annotation and classification tool DAVID (Ashburner et al., 2000; Huang et al., 2009a,b; The Gene Ontology Consortium, 2019). DEGs were enriched in 40 GO terms in “*biological process (BP),”* 16 GO terms in “*cellular component (CC),”* and 22 GO terms in “*molecular function (MF),”* with statistical significance, according to the rule that each gene is annotated with multiple classifications if they match (Supplementary Table 3). Figure 5A shows the top 10 GO terms of BP, top 5 GO terms of CC, and top 5 GO terms of MF, based on the lowest *P*-value rank (Figure 5A). Interestingly, with the BP group, five GO terms (“DNA methylation on cytosine,” “DNA replication-dependent nucleosome assembly,” “positive regulation of gene expression, epigenetic,” “DNA replication-independent nucleosome assembly,” and “chromatin silencing at rDNA”) were significantly altered in BTBR/R mice compared to B6 mice, since many histone protein variant genes including histone H3 (*Hist1h3f*) and H4 (*Hist1h4d, Hist1h4i, Hist1h4j, Hist1h4k, Hist2h4*, and *Hist4h4*) family genes were DEGs of BTBR/R mice (Supplementary Table 3); this suggests a difference in DNA and chromatin regulation between the two mouse strains. It is also remarkable that the GO term with the lowest *P*-value in the CC and MF included a large number of annotated DEGs: 137 DEGs of “extracellular exosome” and 161 DEGs of “metal ion binding,” respectively (Figure 5B). In addition, the enrichment of “*immune signaling”*-related GO terms, 9 in the BP and 2 in the MF, was a characteristic in BTBR/R mice DEGs (Figure 5B).

**Figure 6:**
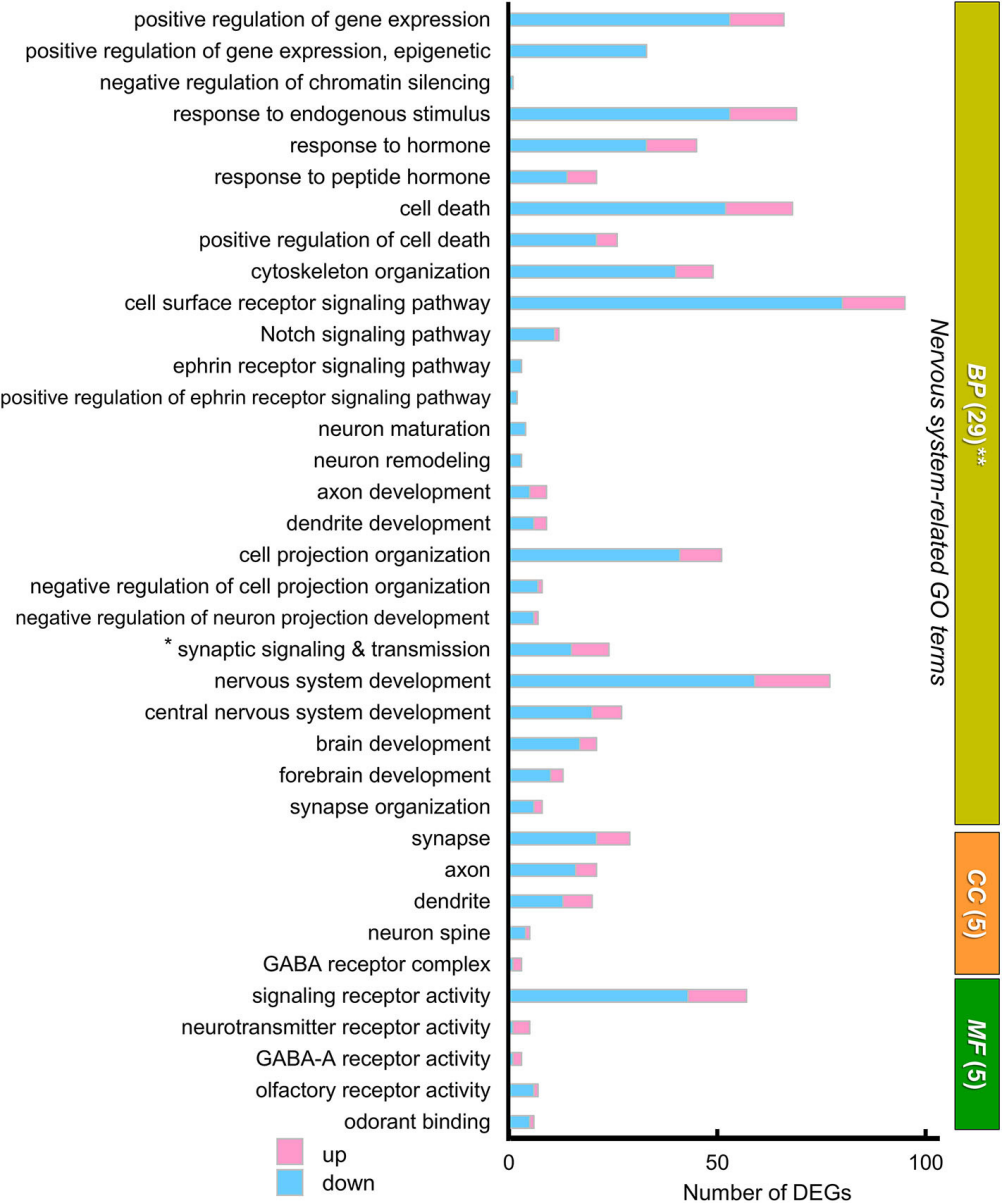
The “nervous system-related” GO terms of DEGs. A graph showing 39 “nervous system-related” GO terms from 29 BP, 5 CC, and 5 MF sub-ontologies. Many DEGs were annotated to a variety of these nervous system related GO terms, although these annotations were not statistically significant. Blue and pink bars represent downregulated and upregulated DEGs, respectively. * “Synaptic signaling and transmission” includes four GO terms: “synaptic signaling,” “trans-synaptic signaling,” “anterograde trans-synaptic signaling,” and “chemical synaptic transmission.” **In BP, the GO term “gene expression” (154 downregulated genes, 39 upregulated genes) is not included in this graph. In this GO annotation, each gene is annotated with multiple terms, if they match, so that number of DEGs shown in the graph of GO terms includes redundancy.

We next focused on “*nervous system”*-related GO terms (Figure 6). In the BP group, “*Cell surface receptor signaling pathway”* (80 down- and 15 up-regulated genes) and “*nervous system development”* (59 down- and 18 up-regulated genes) were the first and second ranked groups, respectively, also suggesting possible changes in cellular signaling and development of the cerebral cortex and striatum between the two strains. Sixteen selected GO terms related to brain development included 199 down- and 66 up-regulated genes (with redundancy), suggesting that these DEGs may influence developmental differences in the cerebral cortex and striatum between the two strains of mouse (Supplementary Table 3).

#### KEGG Pathway Analysis

**Figure 7:**
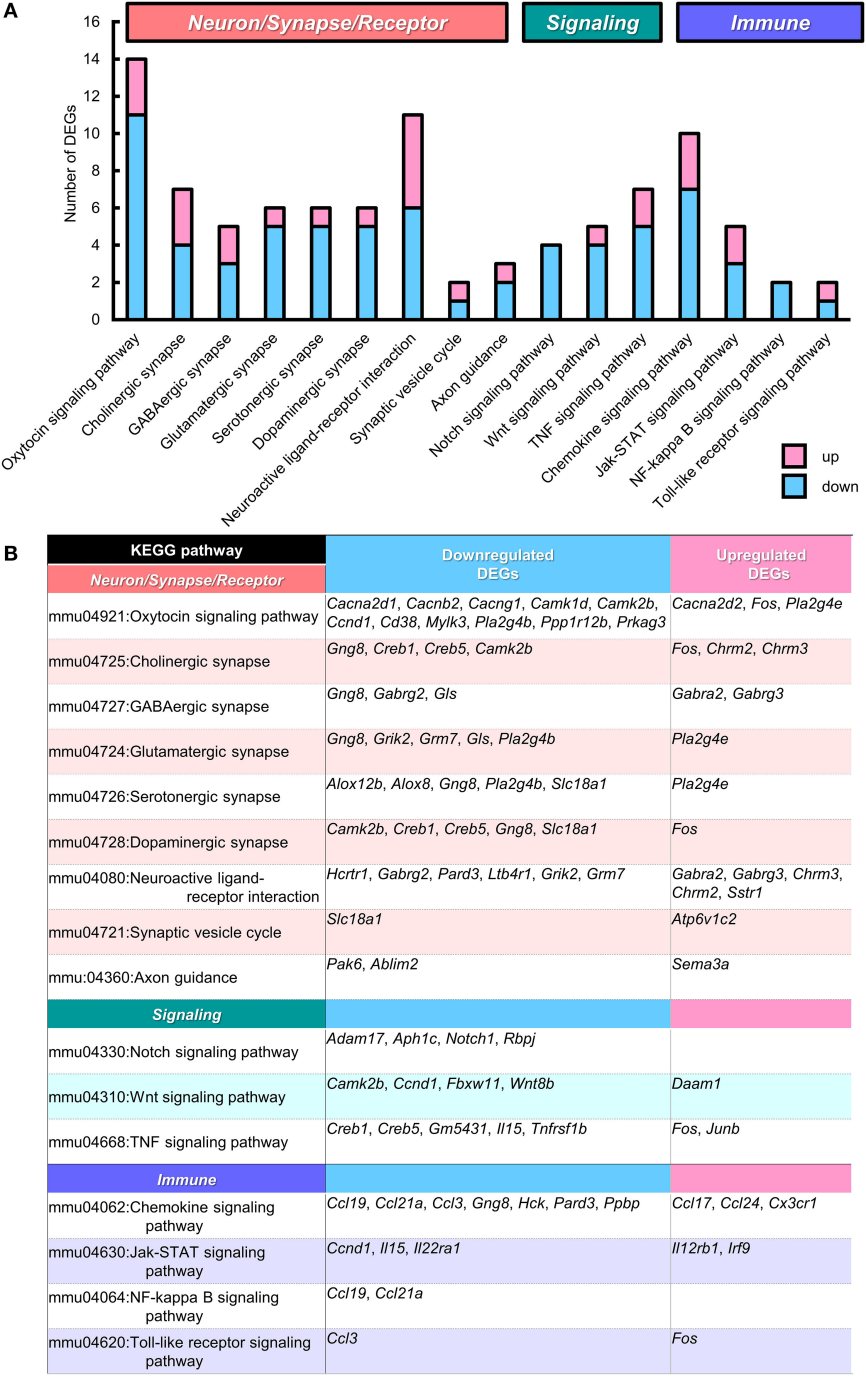
KEGG pathways that suggest functional alterations in BTBR/R. From Kyoto Encyclopedia of Genes and Genomes (KEGG) pathway analysis data (Supplementary Table 4), we selected 15 KEGG pathways which are categorized into the pathways related to “Neuron/Synapse/Receptor” (8 pathways), “Signaling” (3 pathways), and “Immune” (4 pathways). Among these pathways, the “Oxytocin signaling pathway” showed statistical significance (*P* < 0.00253; see Supplementary Table 4). **(A)** Number of differentially expressed genes (DEGs) classified into these 15 pathways (with redundancy) are shown. Blue and pink bars represent downregulated and upregulated DEGs, respectively. **(B)** Gene symbols of DEGs classified into each KEGG pathway.

We next analyzed the functional connection of DEGs using the KEGG pathway database (Kanehisa et al., 2017). The 12 statistically significant pathways (*P* < 0.05) are summarized in Supplementary Table 4. “*Viral carcinogenesis”* (21 DEGs, *P* = 0.0002), “*Alcoholism”* (15 DEGs, *P* = 0.013), and “*Systemic lupus erythematosus”* (12 DEGs, *P* = 0.015) pathways were highly ranked, which may be partly due to the alteration of many histone protein variant genes as described above. Importantly, “*Oxytocin signaling pathway”* (to which 14 DEGs were specified) was included (11 down- and 3 up-regulated, *P* = 0.0025), suggesting the possibility that altered gene expression in this pathway may influence the oxytocin-mediated behavior in BTBR/R mice. To individually assess DEGs mapped to specific pathways regardless of connectivity, we reexamined the KEGG pathway mapping data and picked up 15 pathways (including “*Oxytocin signaling pathway”*) in terms of Neuron/Synapse/Receptor, Signaling, and Immune response from all hit pathways including ones with *P* ≧ 0.05 (Figure 7A and Supplementary Table 4). The results indicated that there were 11 DEGs mapped to “*Neuroactive ligand-receptor interaction pathway”* of which six genes encoded neurotransmitter receptors for GABA (down: *Gabrg2*; up: *Gabra2, Gabrg3*), acetylcholine (up: *Chrm2, Chrm3*), and glutamate (down: *Grm7*; Figure 7B). The genetic alterations in these receptor genes have been shown to contribute to neuropsychiatric disorders, including GABAergic receptors *Gabrg2* (epilepsy), *Gabra2* (alcohol dependence), and *Gabrg3* (developmental delay, ASD, and Prader-Willi/Angelman syndrome; Braat and Kooy, 2015), muscarinic acetylcholine receptor *Chrm3* (ASD; Petersen et al., 2013), and metabotropic glutamate receptor *Grm7* (ASD; Noroozi et al., 2016). This reexamination also showed the alteration of gene expression for three cell response pathways to cell-cell signaling molecules (Notch, Wnt, and TNF). In addition, the enrichment of 10 DEGs in “*Chemokine signaling pathway”* (7 down and 3 up; Figure 7B) was detected, thereby supporting the possibility that the immune signaling is altered between the two mouse strains.

#### WGCNA

**Figure 8:**
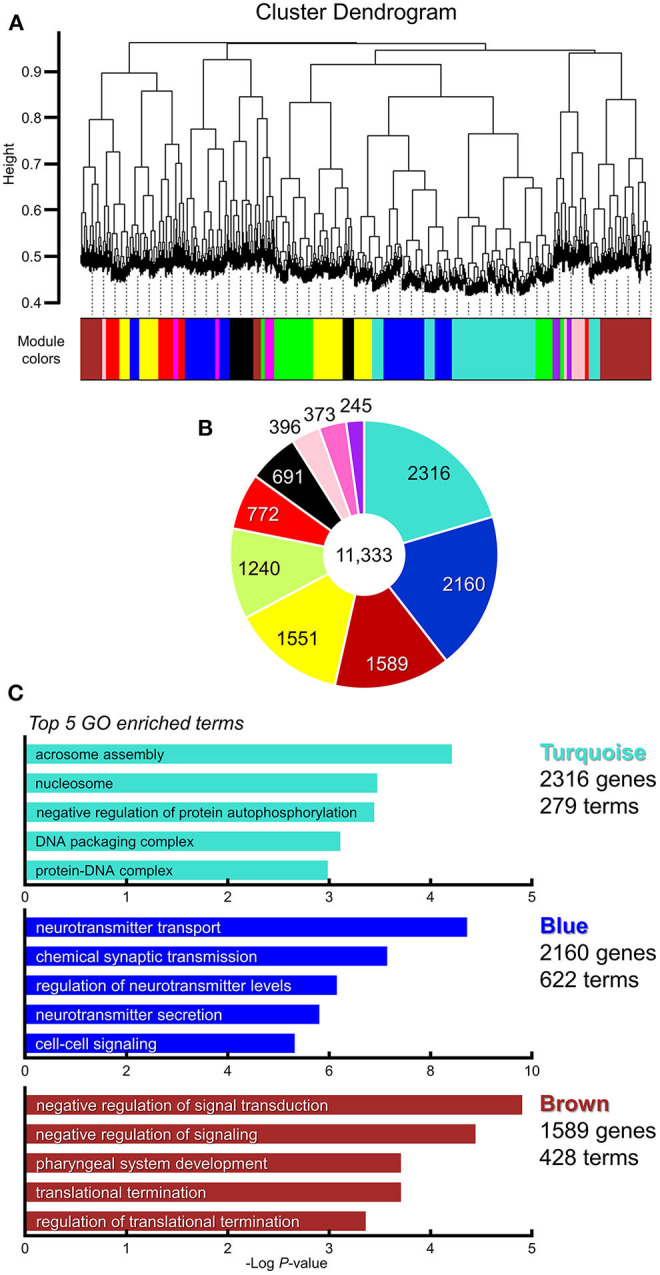
WGCNA identified multiple clusters (modules) of highly correlated genes which were enriched in synapse category. Weighted gene co-expression network analysis (WGCNA). Gene subset containing 11,323 genes is selected for WGCNA analysis (the top 20% of probes; Supplementary Table 5). **(A)** Gene dendrogram and clustered 10 modules coded by different colors. **(B)** Pie chart showing gene counts of each module. **(C)** Top 5 enriched gene ontology categories in modules of the highest three (“Turquoise module,” “Blue module,” and “Brown module”). Horizontal axis indicates the “Log *P*-value” (Enrichment *P*; Supplementary Table 6).

To elucidate gene co-expression networks, we analyzed the microarray sample data by weighted correlation analysis (Zhang and Horvath, 2005; Langfelder and Horvath, 2008). For this analysis, we selected 11,323 probes that were the top 20% most informative probes reliable for the detection of their expression (Provenzano et al., 2016). The WGCNA analysis of BTBR/R data normalized to B6 data showed 10 highly-correlated modules of co-expressed genes (Figures 8A,B, Supplementary Table 5). The top 3 large modules “*Turquoise module”* (2,316 genes; 279 GO terms), “*Blue module”* (2,160 genes; 622 GO terms), and “*Brown module”* (1,589 genes; 428 GO terms) are enriched for genes annotated to the GO terms related to Nucleosome-Chromatin-DNA (“nucleosome,” “DNA packaging complex,” and “protein-DNA complex”), Neurotransmitter-Synaptic Transmission (“neurotransmitter transport,” “chemical synaptic transmission,” “regulation of neurotransmitter levels,” “neurotransmitter secretion,” and “cell-cell signaling”), and Signal Transduction-Translational Regulation (“negative regulation of signal transduction,” “negative regulation of signaling,” “translational termination,” and “regulation of translational termination”), respectively, with the highest statistical significance (Figure 8C, Supplementary Table 6).

Overall, our comprehensive bioinformatics analyses of gene expression highlighted functional alterations in the cerebral cortex and striatum of BTBR/R mice.

### Fifty-Three ASD Candidate Genes Were Included in DEGs Between BTBR/R and B6 Mice

**Figure 9:**
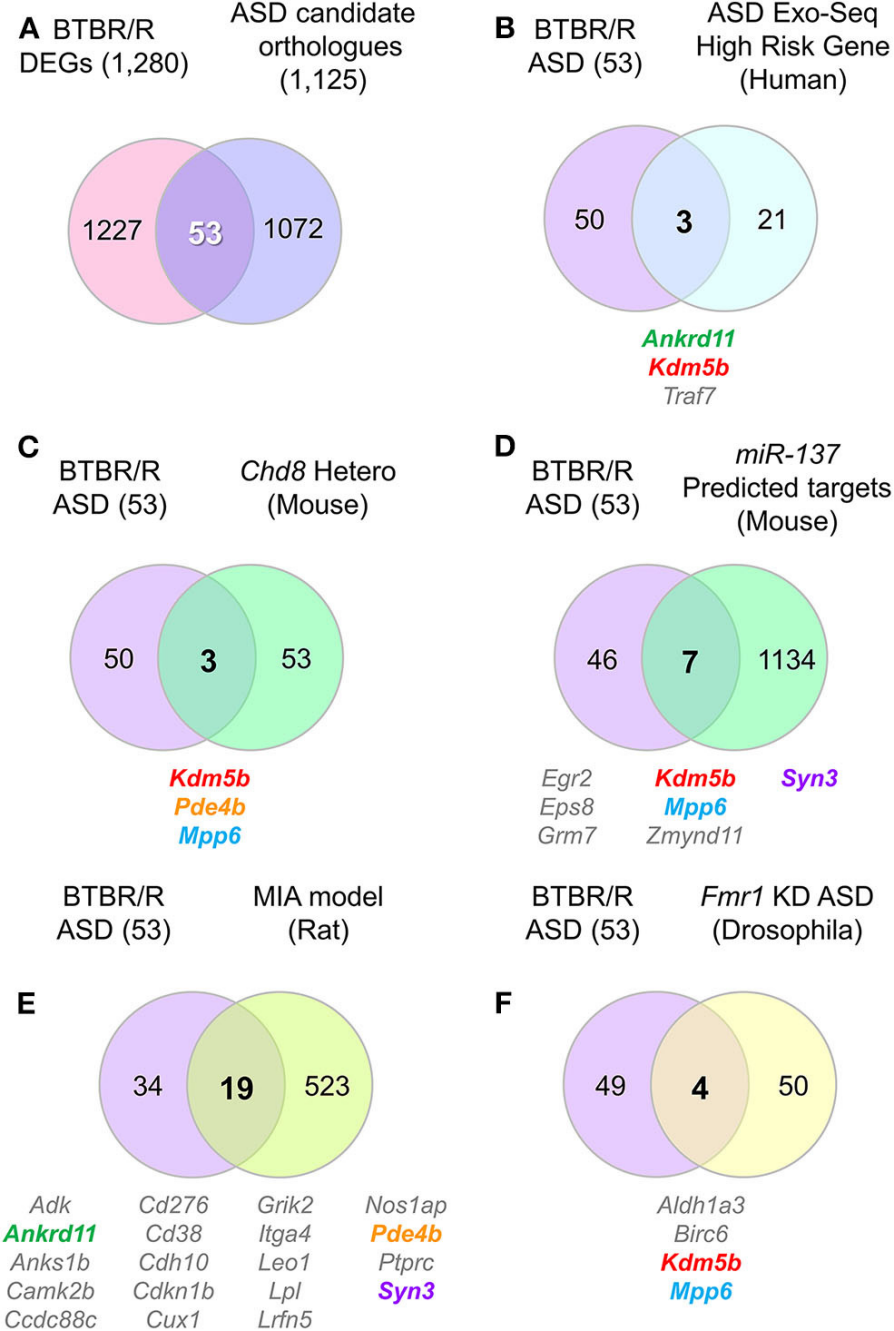
DEG contained several ASD-related orthologs which overlapped with other ASD model and/or human patient altered genes. Venn diagrams show the number of identical genes between BTBR/R differentially expressed genes (DEGs) and autism spectrum disorder (ASD)-related genes registered in the ASD database AutDB **(A)**, and the resultant 53 BTBR/R ASD-related genes and the genes reported in a larger ASD exome sequencing study **(B)** and four ASD model animal studies **(C–F)**. **(A)** 53 genes are identical between 1,280 BTBR/R DEGs and 1,125 ASD-related mouse orthologs to 1,141 human ASD-related genes (AutDB statics, updated Jan 2020). The 53 overlapped genes between BTBR/R DEGs and ASD-related genes are referred to as “BTBR/R ASD genes.” **(B)** There were 3 overlapped genes (*Ankrd11, Kdm5b*, and *Traf7*) between the large ASD exosome sequencing study (Satterstrom et al., 2020) and this BTBR/R study. **(C)** There were also 3 overlapped genes (*Kdm5b, Pde4b*, and *Mpp6*) between the *Chd8* haploinsufficient transgenic mouse study (Suetterlin et al., 2018) and this BTBR/R study. **(D)** There were 7 overlapped genes (*Egr2, Eps8, Grm7, Kdm5b, Mpp6, Zmynd11*, and *Syn3*) between the *miR137* overexpressing transgenic mouse study (Cheng et al., 2018) and this BTBR/R study. **(E)** There were 19 overlapped genes (*Adk, Ankrd11, Anks1b, Camk2b, Ccdc88c, Cd276, Cd38, Cdh10, Cdkn1b, Cux1, Grik2, Itga4, Leo1, Lpl, Lrfn5, Nos1ap, Pde4b, Ptprc*, and *Syn3*) between the lipopolysaccharide-induced maternal immune activation rat fetus ASD model study (Oskvig et al., 2012; Lombardo et al., 2018) and this BTBR/R study. **(F)** There were four overlapped genes (*Aldh1a3, Birc6, Kdm5b*, and *Mpp6*) between the *Fmr1* knockdown *Drosophila* embryo study (Greenblatt and Spradling, 2018) and this BTBR/R study. Five gene symbols that show co-occurrence among multiple independent studies are shown in colored letters; they are: *Ankrd11, Kdm5b, Pde4b, Mpp6*, and *Syn3*.

We next explored whether BTBR/R mice DEGs were the known ASD candidate genes by utilizing the autism gene database AutDB (Pereanu et al., 2018). We identified 53 genes (40 downregulated and 13 upregulated) out of 1,280 DEGs to be ASD candidate genes, which corresponds to about 4.7% of total 1,125 genes registered in the AutDB (Figure 9A, Supplementary Table 9). Considering this result, we entertained the possibility of altered co-expression or combinational expression patterns of these 53 DEGs in BTBR/R mouse brains being partly associated with ASD.

To verify this possibility further, we next analyzed the co-occurrence of differentially-expressed ASD candidate genes between BTBR/R and B6 mice (hereafter referred to as “BTBR/R ASD-DEGs”) in the previous studies of three other psychiatric disorder models and human subjects with ASD (Figures 9B–F, Supplementary Tables 12, 13). Comparing with the genes affected in ASD patients (the largest genome-scale exome-sequencing meta-analysis [*n* = 35,584 total samples, 11,986 with ASD] by Satterstrom et al., 2020; Figure 9B), *Chd8* (chromodomain helicase DNA binding protein 8: ASD-candidate gene) haploinsufficient transgenic mice (with increased connectivity in cortical network and no obvious sociability deficits; P5 mice; one brain hemisphere; Suetterlin et al., 2018; Figure 9C), *miR137* haploinsufficient transgenic mice (with synaptic overgrowth, dendritic growth deficits, learning and memory deficits and social behaviors deficits; P14 mice; whole brain; Cheng et al., 2018; Figure 9D), the lipopolysaccharide (LPS)-induced maternal immune activation (MIA) rat fetus model (with neural and behavioral abnormalities relevant to ASD; LPS manipulation for the MIA-inducing event on gestational day 15, gene expression measured at 4 h post-LPS injection; Oskvig et al., 2012; Lombardo et al., 2018; Figure 9E), and *Fmr1* (Fragile X mental retardation 1: ASD-candidate gene) knockdown *Drosophila* embryos (with neural defects; stage 14 follicles; Greenblatt and Spradling, 2018; Figure 9F). The results allowed us to verify *de novo* variations of three ASD-associated BTBR/R DEGs (*Ankrd11, Kdm5b*, and *Traf7*) in the large exome sequencing study of patients with ASD (Satterstrom et al., 2020; Figure 9B and Supplementary Table 13). Similarly, the co-occurrence of BTBR/R ASD DEGs was 3 in the *Chd8* study (Figure 9C), 7 in the *miR137* study (Figure 9D), 19 in the MIA study (Figure 9E), and 4 in the *Fmr1* study (Figure 9F). It was remarkable that five BTBR/R ASD genes repeatedly co-occurred in multiple independent studies: *Kdm5b* in all four studies; *Mpp6* in three studies; and *Ankrd11, Pde4b*, and *Syn3* in two studies. In addition, 19 out of 53 BTBR/R ASD DEGs co-occurred in the MIA model study. These results suggested that BTBR/R mouse brains have alterations in *Kdm5b*-mediated epigenetic regulation commonly affected in these ASD models together with those in the maternal immune activation model.

We next examined DEGs between two sublines of BTBR mice by comparing our BTBR/R vs. B6 dataset (1,280 DEGs obtained from the cerebral cortex and striatum) with the five independent BTBR/J vs. B6 datasets (1,016 DEGs from the hippocampus by Provenzano et al. (2016); 325 DEGs from the cerebellum by Shpyleva et al. (2014); 448 DEGs in the striatum by Oron et al. (2019); and 328 DEGs from the hippocampus and 328 DEGs from the cerebral cortex by Daimon et al., 2015; Supplementary Table 14). The 341 DEGs identified in our BTBR/R study were also reported as DEGs in at least one of the BTBR/J datasets. Five DEGs showed consistent expression patterns in all five datasets: three upregulated DEGs (*Adi1, Scg5*, and *Serpina3n*), and two downregulated DEGs (*Nudt19* and *Pop4*). The expression patterns of 136 DEGs (23 upregulated, 113 downregulated) were consistent between the BTBR/R mice cerebral cortex and striatum and the BTBR/J mice striatum (Oron et al., 2019) datasets, while those of 30 DEGs (6 upregulated, 24 downregulated) were consistent between the BTBR/R mice cerebral cortex and striatum and the BTBR/J mice cerebral cortex (Daimon et al., 2015) datasets (Supplementary Table 14). In total, 208 DEGs showed consistent expression patterns (45 upregulated, 163 downregulated) between the BTBR/R cerebral cortex and striatum and BTBR/J hippocampus (Provenzano et al., 2016) datasets. The results suggest both differences and similarities in the brain transcriptomes of BTBR/R and BTBR/J mice, although well-controlled direct comparisons between the two sublines of BTBR are required.

### Spatial Expression Patterns of DEGs Were Also Different Between B6 and BTBR/R Brain

**Figure 10:**
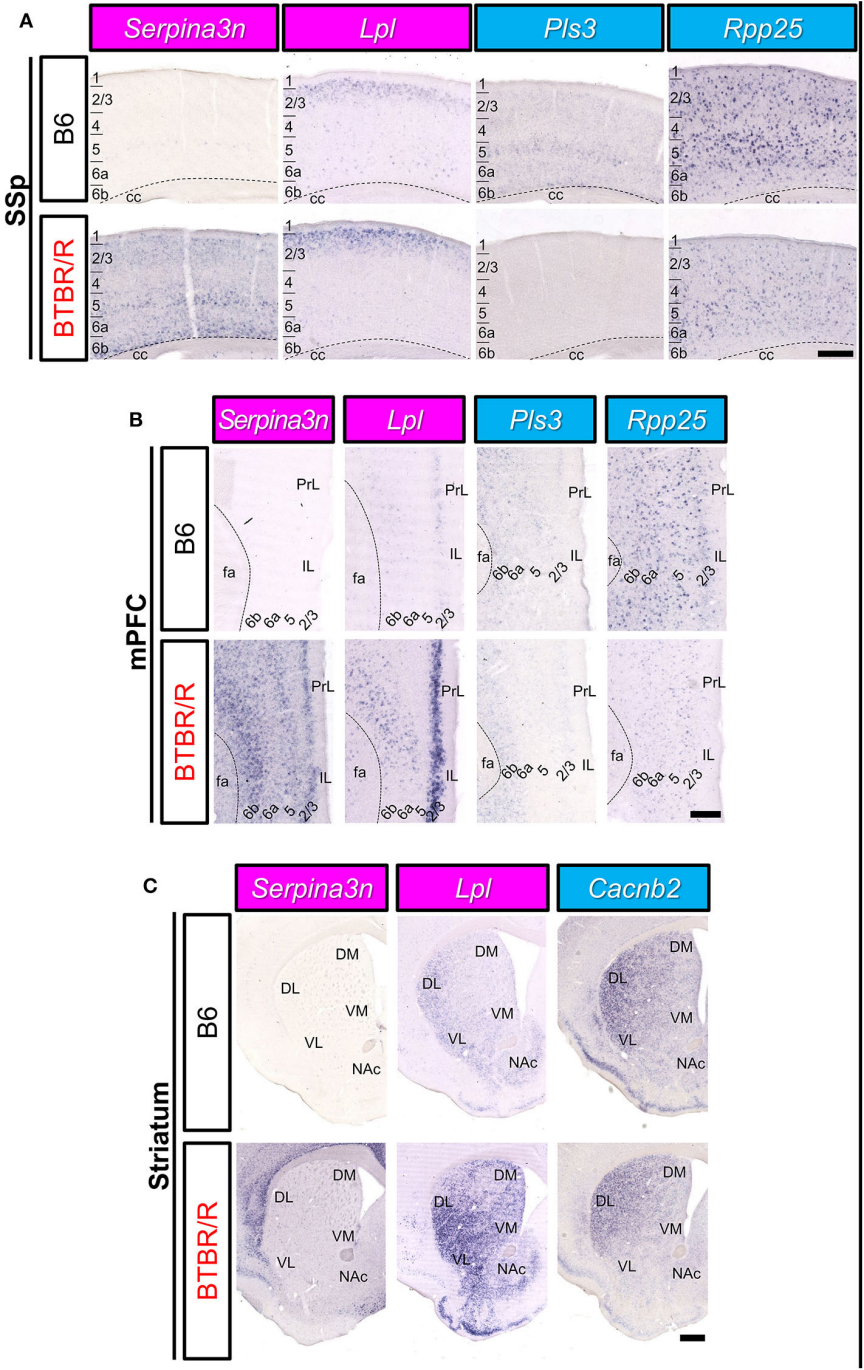
The spatial expression of DEGs in the cerebral cortex and striatum of B6 mice and BTBR/R mice. *In situ* hybridization images for gene expression of *Serpina3n, Lpl, Pls3, Rpp25*, and *Cacnb2* in coronal sections of the primary somatosensory cortex (SSp; **A**), medial prefrontal cortex (mPFC; **B**), and striatum **(C)**. Upper and lower columns show B6 and BTBR/R mouse sections, respectively. Scale bars show 250 μm in cortical images and 500 μm in the striatum images. 1, 2/3, 4, 5, 6a, and 6b, cerebral cortical layer 1, 2/3, 4, 5, 6a, 6b; cc, corpus callosum; fa, corpus callosum, anterior forceps; DL, dorsolateral striatum; DM, dorsomedial striatum; IL, infralimbic cortex, NAc, nucleus accumbens; PrL, prelimbic cortex; VL, ventrolateral striatum; VM, ventromedial striatum. The magenta and light blue indicate upregulated and downregulated genes, respectively.

We comprehensively analyzed the spatial cellular expression of DEGs in the BTBR/R and B6 mice brains in a qualitative manner using the ISH method. We selected 11 DEGs (3 upregulated and 8 downregulated) which are well-annotated in GO. Consistent with microarray analysis, the expression level of *Serpina3n* and *Lpl* in cortical areas and the striatum was higher in BTBR/R mice than B6 mice (Figure 10). Higher expression of *Serpina3n* in BTBR/R mice was entirely observed in cortical layers of the primary somatosensory cortex (SSp; Figure 10A) and medial prefrontal cortex (mPFC; Figure 10B), but the difference between BTBR/R and B6 mice was not prominent in the striatum (Figure 10C). Interestingly, the strong expression of *Lpl* in BTBR/R mice was observed in the superficial layer of cortex (Figures 10A,B). In the striatum, *Lpl* was highly expressed in BTBR/R compared with B6 mice, especially in the ventral lateral striatum (VL; Figure 10C). In contrast, the cortical expression of *Pls3* and *Rpp25* in BTBR/R mice was lower than that of the B6 mice (Figures 10A,B). The expression of *Cacnb2* in the nucleus accumbens was decreased in BTBR/R compared with B6 mice (Figure 10C). The expression of other DEGs in the cerebral cortex and striatum are shown in the Supplementary Figures 2–5. Overall, the expression of 11 DEGs in the cortex and/or striatum from ISH was consistent with the results from the microarray and qRT-PCR analyses (Figure 10, Supplementary Figures 2–5).

**Figure 11:**
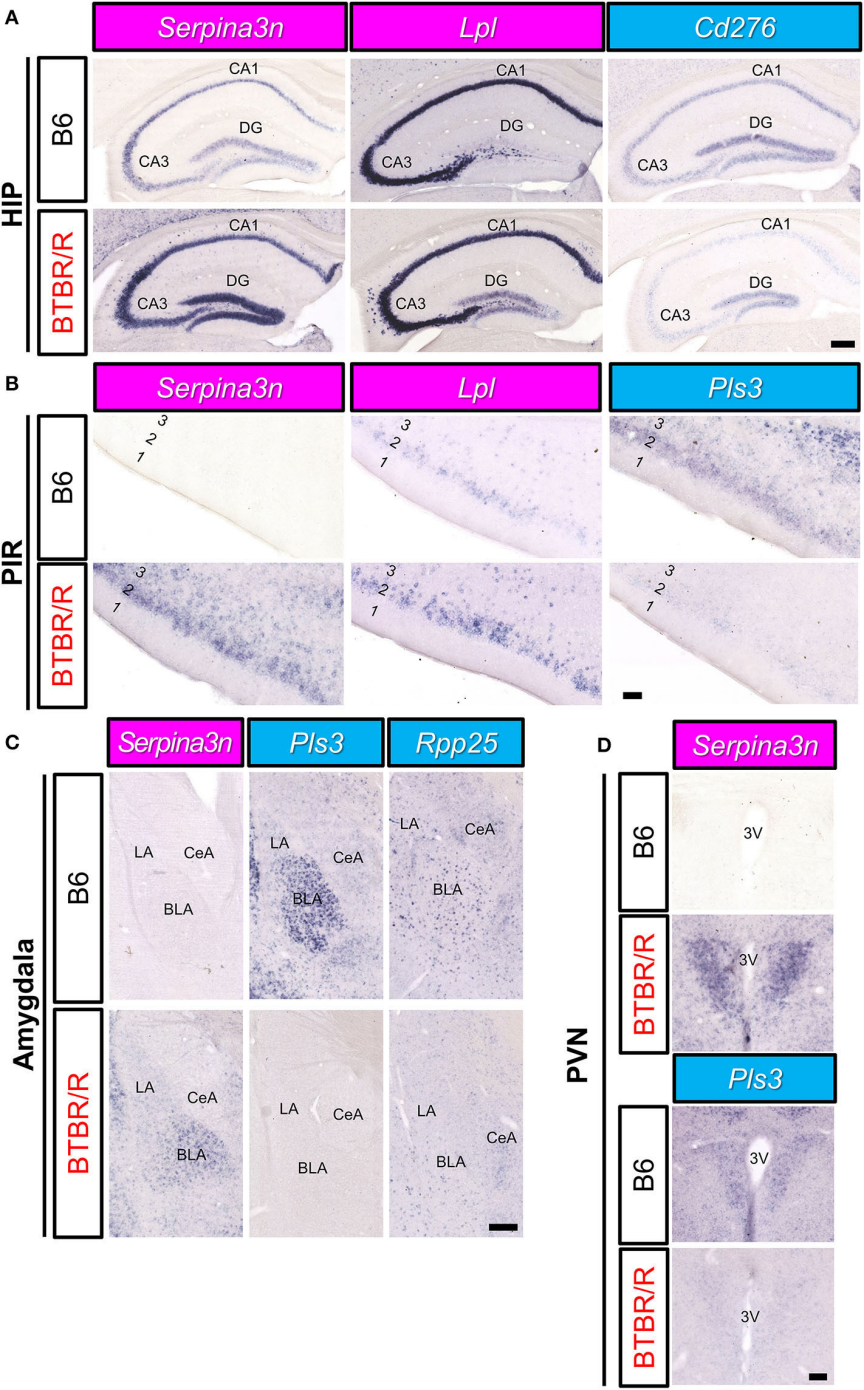
The spatial expression of DEGs in the hippocampus, amygdala, and PIR of B6 mice and BTBR/R mice. *In situ* hybridization images for gene expression of *Serpina3n, Lpl, Cd276, Pls3*, and *Rpp25* in coronal sections of the hippocampus (HIP; **A**), piriform cortex (PIR; **B**), amygdala **(C)**, and paraventricular nucleus (PVN; **D**). Upper and lower columns show B6 and BTBR/R mouse sections, respectively. Scale bars show 250 μm in the HIP and amygdala, 100 μm in the PIR and PVN. In the HIP, CA1, pyramidal layer of the cornu ammonis 1; CA3, pyramidal layer of the cornu ammonis 3; DG, dentate gyrus. In the amygdala, BLA, basolateral amygdala; CeA, central amygdala; LA, lateral amygdala. In the PIR, 1, 2 and 3, cerebral cortical layer 1, 2, and 3. In the PVN, 3V, third ventricle. The magenta and light blue indicate upregulated and downregulated genes, respectively.

Next, we analyzed the expression of DEGs in other brain areas (Figure 11). Similar to the cortical areas, *Serpina3n* expression of BTBR/R mice was higher than that of B6 mice in the hippocampal CA1, CA3, and dentate gyrus (DG) areas (Figure 11A). In BTBR/R mice, the expression of *Lpl* was specifically increased in the DG compared with B6 mice (Figure 11A). In contrast, the expression of *Cd276* in the CA1 and CA3 areas was lower in BTBR/R than B6 mice (Figure 11A). In the piriform cortex (PIR), the expression of *Serpina3n* and *Lpl* was increased, while that of *Pls3* was decreased, in BTBR/R compared to B6 mice (Figure 11B). In the amygdala, the expression of *Serpina3n* was higher in BTBR/R than B6 mice, especially in the basolateral amygdala (BLA) (Figure 11C). By contrast, the expression of *Pls3* was lower in BTBR/R than B6 mice in the BLA (Figure 11C). Consistent with the cortical areas, *Rpp25* expression of BTBR/R mice was decreased in the amygdala compared with that of B6 mice (Figure 11C). The expression of other DEGs in the hippocampus, PIR, and amygdala is shown in Supplementary Figures 6–8. Finally, we focused on the paraventricular hypothalamic nucleus. The expression of *Serpina3n* and *Pls3* was increased and decreased in BTBR/R mice compared to B6 mice, respectively (Figure 11D).

**Figure 12:**
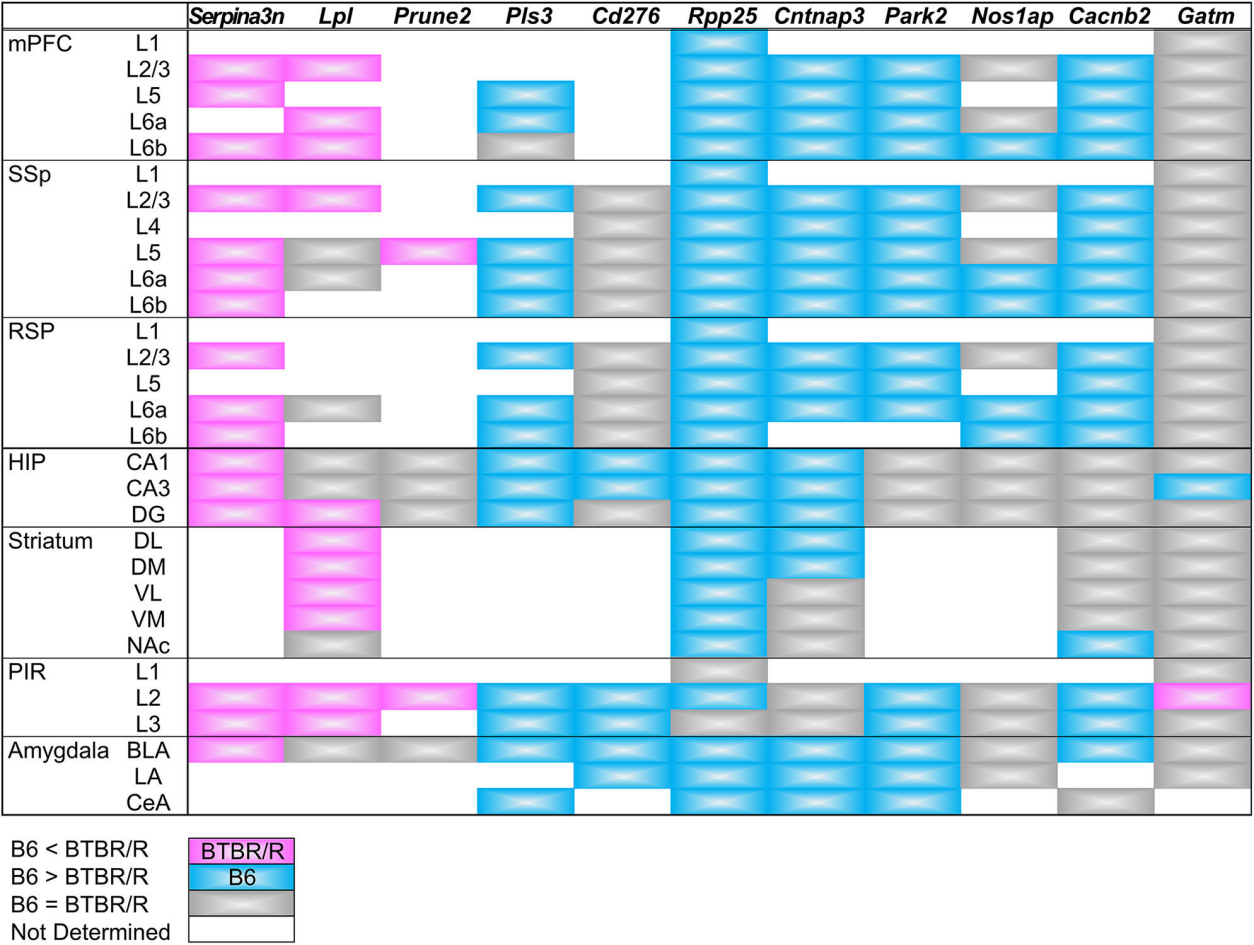
Summary of DEGs expression in the cortical and subcortical areas. Comparative expression levels of differentially expressed genes between BTBR/R mice and B6 mice are shown by color. Magenta and cyan indicate that the *in situ* hybridization signal level is higher in BTBR/R mice and B6 mice than B6 mice and BTBR/R mice, respectively. Gray indicates no detectable difference between two mouse strains. Abbreviations are indicated in the legends of Figures 10, 11.

We have summarized the spatial pattern of DEG expression in Figure 12. Essentially, messenger RNA (mRNA) expression levels detected by ISH were consistent with the results indicated by the microarray and qRT-PCR analyses. In addition, the comparative ISH analysis provided valuable data on the cellular and regional difference of DEG expression between BTBR/R and B6 mouse brains, which is informative for future studies to consider a relationship between gene expression and brain circuits associated with pathologies.

### BTBR/R Behavioral Phenotypes Were Indicative of a Slight Autistic Tendency

Finally, we assessed the basic phenotypes of BTBR/R mice in terms of sociality and associated emotional features. In five-trial social habituation/recognition tasks (Supplementary Figure 9A), compared to B6 mice (*n* = 12), BTBR/R mice (*n* = 15) unexpectedly showed no significant differences in four repeated habituation interaction trials with the first social stimulus mouse (Supplementary Figure 9B). Interestingly, response to a second novel stimulus mouse at trial five was slightly decreased in BTBR/R compared to B6 mice (Supplementary Figure 9B, repeated measurement two-way ANOVA; trial 4–5, *F*_(1, 25)_ = 35.80, *P* < 0.001; mouse strains, *F*_(1, 25)_ = 0.15, *P* = 0. 70; trials × strain, *F*_(1, 25)_ = 4.31, *P* = 0.048; *post-hoc* Tukey-Kramer test, BTBR/R vs. B6 at trial 5, *P* = 0.026). Then, we analyzed the social recognition index in which interaction time with a novel mouse at trial five was subtracted from that with a habituated mouse at trial four. The social recognition index was significantly decreased in BTBR/R compared with B6 mice (Supplementary Figure 9C, *P* = 0.048). In open field tests (Supplementary Figures 9D–G), BTBR/R mice showed a striking increase in repetitive jumping behavior compared with B6 mice (*P* < 0.001), although there were no differences in total distance, time spent in center area, and time spent self-grooming. These findings suggest that, compared to B6 mice, BTBR/R mice have a mild impairment in social recognition as well as increased stereotypic behavior.

## Discussion

In this study, we identified a number of transcriptomic changes in the cerebral cortex and striatum of BTBR/R mice (1,280 DEGs with 974 downregulated and 306 upregulated alterations) compared to B6 mice. The GO enrichment analysis showed 78 GO annotations and highlighted the significant alteration of functional gene groups including the DNA-chromatin-related gene group, which may influence DNA replication, transcriptional and epigenetic regulation, and the immune signaling-related gene group, which may result from increased and/or aberrant immune responses. The KEGG pathway analysis showed enrichment of the “*Oxytocin signaling pathway”*-related 14 DEGs with 11 downregulated and three upregulated expression profiles, suggesting the possibility of an overall decline in this important pathway to express the oxytocin-mediated social behavior. The WGCNA co-expression network analysis indicated significant changes in the co-expression modules including a large number of DNA/chromatin-related genes, synaptic transmission-related genes, and signal transduction/translational regulation-related genes. We also showed that 53 ASD-related genes have differential expression patterns between BTBR/R and B6 mice brains. By comparing our DEGs and these ASD-related DEGs to DEGs reported by five independent studies of ASD patients and ASD animal models, we propose some gene candidates critical to similar phenotype(s) among ASD patients and ASD animal models. Moreover, by comparing our DEGs to DEGs reported in four independent BTBR/J studies, we show differences and similarities between the two BTBR sublines, and suggest some critical DEGs that commonly influence brain function and behavior. Finally, we mapped spatial differential expression patterns of 11 DEGs in BTBR/R mice in comparison to those of B6 mice. These transcriptomic features in the cerebral cortex and striatum of BTBR/R mice in contrast to those of highly social B6 mice suggest alterations in expression of brain functions and behavior between these two mouse strains.

### RNA Alterations in BTBR/R Mice: ncRNA Expression, Splicing, and RNA–RNA Interactions

In addition to alteration of protein-coding transcripts, abnormalities in splicing patterns as well as ncRNA profiles have been shown in individuals with various psychiatric disorders including ASD (Gandal et al., 2018). Specific ncRNA species such as micro RNAs (miRNAs), small nucleolar RNAs, and lncRNAs are involved in psychiatric disorders by regulating transcriptional and translational systems (Zhang et al., 2019). Abnormal alternative splicing is also associated with neurodevelopmental disorders (Zhang et al., 2016; Gandal et al., 2018); for instance, Rbfox1 is a transcription regulator that controls alternative splicing and its mutation is associated with ASD (Wamsley et al., 2018). In addition, the alternative splicing “Retained intron” is involved in increasing miRNA targets by changing untranslated region's sequence in ASD-related genes (Tan et al., 2007). In this study, we identified 498 ncRNAs (387 downregulated, 111 upregulated) differentially expressed in BTBR/R mice compared to B6 mice. In addition, it was notable that 18.2% of BTBR/R mice DEGs showed alternative splicing (160 downregulated, 47 upregulated; “nonsense mediated decay” and “retained intron”). The altered ncRNA and alternative spliced transcript expression may contribute to a transcriptomic feature in BTBR/R mice brain.

In this study, we identified a huge RNA–RNA interaction network that included DEG- and ASD-related genes. As mentioned above, ncRNA have a regulatory role via ncRNA–RNA interactions. In addition, recent studies have shown that mRNA-mRNA interactions also act as translational regulators in prokaryotes (Ruiz de los Mozos et al., 2013; Masachis and Darfeuille, 2018; Ignatov et al., 2020). In our analysis, we identified *Gm37194* and *Gm26917* as a hub gene that was the most connected node with other node RNAs. Both *Gm37194* and *Gm26917* are predicted genes annotated by an expressed sequence tag (EST; Wilming et al., 2008). *Gm26917* lncRNA expression is regulated by FoxM1 and acts as a competing endogenous RNA source for miRNA-29b, which accelerates apoptosis of muscle satellite cells (Chen et al., 2018). We also identified *Ankrd11* and *Cadps2* as the most connected genes of all the ASD-related genes. ANKRD11 is a potential chromatin regulator implicated in neural development, and its *de novo* mutation was reported in ASD (Iossifov et al., 2014). In mice, ANKRD11 controls cortical precursor proliferation via histone acetylation, and its knockdown mice showed ASD-like behavior (Gallagher et al., 2015). CADPS2 is a cytosolic protein that regulates the exocytosis of synaptic and dense-core vesicles (Cisternas et al., 2003). In ASD patients, an aberrant increase of a rare alternative splicing variant with exon3-skipping was reported and mice expressing exon3-skipped *Cadps2* variant showed ASD-like behavior (Sadakata et al., 2007, 2012). Among *Ankrd11*-interacting ASD-related genes, six transcriptional repressors (*Phf12, Tbr1, Rere, Ctcf*, *Ep400*, and *Per1*) and three E3 ubiquitin protein ligases (*Ube3b, Trip12*, and *Ubr5*) were included. It is of interest that within the *Cadps2*-interacting DEGs, there were three RNA splicing related genes (*Cdk13, Srrm2*, and *Sfpq*). Taken together, the RNA–RNA interaction network data demonstrates that *Ankrd11* and *Cadps2* interact with many functionally-related genes, suggesting that these functional mRNA-mRNA interactions may underlie the transcriptional regulation of ASD-related genes.

### BTBR/R DEGs Are Commonly Altered in ASD Individuals and Animal Models

We identified 53 ASD candidate genes within BTBR/R mice DEGs (referred to as BTBR/R ASD-DEGs in this study), suggesting that the alteration of this combinatory gene expression pattern may contribute to differences between BTBR/R and B6 mice. Thus, we further analyzed the co-occurrence of BTBR/R mice ASD DEGs in the previously reported five independent studies: in human ASD patients and animal models (mouse, rat, and *Drosophila*) five BTBR/R ASD-DEGs were found that were repeatedly reported in the previous five studies: *Kdm5b* (four times); *Mpp6* (three times); *Ankrd11, Pde4b*, and *Syn3* (twice). *Kdm5b* was particularly interesting because of co-occurrence in four independent datasets obtained by analyzing human patients with ASD (Satterstrom et al., 2020) and ASD animal models, including *Chd8*-haploinsufficient transgenic mice (Suetterlin et al., 2018), *miR137*-haploinsufficient transgenic mice (Cheng et al., 2018), and *Fmr1*-knockdown *Drosophila* embryos (Greenblatt and Spradling, 2018). KDM5B (lysine demethylase 5B) is one of the lysine-specific histone demethylase family and demethylates tri/di/mono-methylated lysine-4 of histone H3 (H3K4), which is critical to neural development (Schmitz et al., 2011; Fueyo et al., 2015). *De novo* mutations of *Kdm5b* result in a recognizable syndrome with developmental delay (Faundes et al., 2018). MPP6 (membrane protein, palmitoylated 6) is a member of the peripheral membrane-associated guanylate kinase (MAGUK). MPP6 interacts with 4.1G, Lin7, and CADM4 proteins in Schwann cells and its KO mice showed hypermyelination of sciatic nerves (Saitoh et al., 2019). A *de novo* mutation of *Mpp6* was reported in an individual with ASD (Iossifov et al., 2014). Although MPP2, a member of the MPP family, acts as a scaffold in the postsynaptic density of hippocampal CA1 neurons (Kim et al., 2016), the role of MPP6 in the central nervous system remains to be studied. PDE4B is a member of the cyclic nucleotide phosphodiesterase (PDE) family (PDE4 subfamily). A *de novo* synonymous mutation in *PDE4B* was reported in an individual with ASD (Iossifov et al., 2014; Takata et al., 2016). *PDE4B* has also been identified as one of the subset genes related to a common molecular signature in autism (Diaz-Beltran et al., 2016). SYN3 (Synapsin 3) is a synaptic vesicle associated protein and its KO mice showed impairments in early axon outgrowth and inhibitory neurotransmission in hippocampal neurons (Feng et al., 2002). A *de novo* mutation in *SYN3* was identified in an individual with ASD (Ruzzo et al., 2019). TRAF7 (TNF receptor-associated factor 7), which generally exerts negative control on its targets including NF-κB and p53 by ubiquitination, was identified only in the human ASD dataset (Satterstrom et al., 2020). *TRAF7* mutations were shown to be involved in genesis of human cancer, especially in about 25% of meningiomas (Zotti et al., 2017), and *de novo* missense variants were also identified in ASD probands (Neale et al., 2012; Krumm et al., 2015; Tokita et al., 2018). Together, our results suggest that BTBR/R mice have alterations in the *KDM5B, MPP6, ANKRD11, PDE4B, SYN3*, and *TRAF7*-associated pathway(s), some of which are commonly affected in ASD individuals and animal models, that may impact on the development and function of BTBR/R brain.

Maternal infection is a risk for ASD and animal models showed that maternal immune activation is sufficient to impact neuropathology and altered behaviors in offspring (Ehninger et al., 2012; Estes and McAllister, 2015). Recent studies also showed marked neuroinflammation in individuals with ASD, suggesting that can also contribute to ASD risk (Gottfried et al., 2015; Gładysz et al., 2018). Importantly, in addition to changes in gene expression patterns related to the immune signaling and response, which was repeatedly suggested by the GO enrichment and pathway analyses, about 36% of BTBR/R mice ASD DEGs overlapped with DEGs of the LPS-induced MIA rat fetus model (Lombardo et al., 2018). Thus, BTBR/R mice brains may have alterations in the immune-related pathway(s) which impact on brain development and function.

### Similarity in DEGs Between Two Sublines of BTBR: BTBR/R and BTBR/J

By comparison of the DEGs between our BTBR/R study and those of previous BTBR/J studies, we identified five DEGs that commonly appeared among these independent studies (upregulated *Adi1, Scg5*, and *Serpina3n*; downregulated *Nudt19* and *Pop4*), besides the use of different experimental conditions such as brain regions analyzed (cerebral cortex and striatum in this study; hippocampus in Provenzano et al., 2016; cerebellum in Shpyleva et al., 2014; striatum in Oron et al., 2019; and cortex and hippocampus in Daimon et al., 2015). *Adi1* encodes 1,2-dihydroxy-3-keto-5-methylthiopentene dioxygenase that is involved in methionine salvage: 5′-methylthioadenosine cycle to increase S-adenosylmethionine levels, which altered genome-wide promoter methylation profiles, resulting in altered gene expression in hepatocellular carcinoma (Chu et al., 2019). *Scg5* (secretogranin V, 7B2) encodes a secreted chaperone protein that prevents the aggregation of other secreted proteins inside of secretory vesicles, and its KO mice show a number of endocrine abnormalities (Bartolomucci et al., 2011). *Serpina3n* (Serine protease inhibitor A3N) encodes a secretory serine protease inhibitor that mediates neuroinflammation (Xi et al., 2019) and is upregulated in various neurological diseases (Switonski et al., 2015; Vanni et al., 2017). *Nudt19* (Nucleoside diphosphate linked moiety X [Nudix]-type motif 19) encodes a peroxysomal nudix hydrolase (Carreras-Puigvert et al., 2017) that exerts a CoA diphosphohydrolase activity in the kidney (Shumar et al., 2018) and its KO mice caused a significant decrease in total CoA levels in the kidney (Shumar et al., 2018). Neuron-specific overexpression of another peroxisomal CoA hydrolase Nudt7 induced reduction in motor coordination (Shumar et al., 2015). *Pop4* (*Rpp29*) encodes ribonuclease P protein subunit P29 that generates mature transfer RNA (tRNA) by cleaving the 5′-leader sequence from the precursor. A recent study showed that Pop4 represses nucleosome deposition of histone H3.3, a regulator of transcription-state change and epigenetic inheritance (Newhart et al., 2016). Although these five DEGs were repeatedly identified in five independent datasets in two BTBR sublines, BTBR/R and BTBR/J, they are not assigned to ASD candidate genes, and their functions may be important for proper development and function of the human brain and thus remain be elucidated.

### Altered Spatial Expression Patterns of DEG mRNAs in the BTBR/R Mouse Brain

We mapped mRNA expression of 11 DEGs on BTBR/R mice brains. Three upregulated DEGs (*Serpina3n, Lpl*, and *Prune2*) and eight downregulated DEGs (*Pls3, Cd276, Rpp25, Cntnap3, Park2, Nos1ap, Cacnb2*, and *Gatm*) showed similar changing patterns throughout brain regions of BTBR/R and B6 mice. Strikingly, *Serpina3n* was recurrently identified as an upregulated DEG by this BTBR/R study as well as four independent BTBR/J studies, as described above, although it has never been reported as an ASD candidate gene. BTBR/R mice brains showed increased *Serpina3n* mRNA expression: intensely in the cerebral cortex, hippocampus, and amygdala; weakly in the striatum. Together with the transcriptomic data showing the enrichment of the immune signaling pathways in BTBR/R mice DEGs, we suggest that these brain regions in BTBR/R mice may have increased neuroinflammatory responses, as reported in other neurological disorders (Switonski et al., 2015; Vanni et al., 2017; Xi et al., 2019). Upregulated DEG *Lpl* encodes lipoprotein lipase that is the key enzyme in triglyceride metabolism. Highly-increased levels of mRNA expression were remarkable in the cortical layer II/III and striatum of BTBR/R mice compared to B6 mice. LPL activity is critical to regulation of energy balance in the brain (Wang and Eckel, 2012) and is also suggested to play an important role in learning and memory (Xian et al., 2009). Increased expression of *Lpl* in BTBR/R mice brains may cause imbalance of triglyceride-rich plasma lipoproteins, leading to impaired brain function. *Pls3* (*Plastin 3*) encodes an actin binding protein that reduced in a mouse model of spinal muscular atrophy, and its overexpression restored axonal outgrowth of motor neurons in SMA (Alrafiah et al., 2018). *Pls3* mRNA was localized in the cortical layers II/III, V, VIa, VIb, and BLA in B6 mice, but its expression was downregulated in BTBR/R mice, suggesting that BTBR/R mice may have an axonal defect in these regions. *Rpp25* (ribonuclease P and MRP subunit p25) encodes 25 kDa subunit of the ribonuclease P complex and was shown to decrease by about 45% in GABAergic interneurons of the PFC in subjects with ASD (Huang et al., 2010). Importantly, our ISH data also showed a decrease of *Rpp25* mRNA in the mPFC and SSp, probably interneurons, of BTBR/R mice compared to B6 mice, suggesting a possibility that BTBR/R mouse brains may have similar cortical dysfunction to that seen in ASD. Together with *Rpp25*, another key molecule *Pop4* (*Rpp29*) of the ribonuclease P complex is also downregulated in BTBR/R mice as described above, again suggesting a possible defect in tRNA maturation and/or nucleosome formation in these brain regions of BTBR/R mice.

### Two BTBR Sublines: BTBR/R vs. BTBR/J

Both sublines BTBR/R and BTBR/J probably originated from the same BTBRTF/Art line and are bred in the RBRC and the Jackson Lab: in this study we conventionally called them BTBR/R and BTBR/J, respectively. We analyzed the brain transcriptome (DEGs, co-expression and interaction RNA networks) of BTBR/R mice and compared them with data reported in the previous studies of human ASD subjects and ASD animal models, including another BTBR subline of BTBR/J mice. There is one previous study showing enhanced turnover of dendritic spines in the anterior frontal cortex of BTBR/R mice similarly to that seen in ASD model mice during the postnatal developmental stage (Isshiki et al., 2014). On the other hand, there has been accumulating information on BTBR/J mice regarding their genetics, neuropathology, and behavior. Many other hallmark symptoms of ASD have been reported in BTBR/J mice, including low sociability (Bolivar et al., 2007; Moy et al., 2007), resistance to change (Moy et al., 2007, 2008), increased repetitive self-grooming behavior (Pobbe et al., 2010), other repetitive behaviors (Pearson et al., 2011), and reduced territorial scent marking (Wöhr et al., 2011). Our behavioral data provide, for the first time, evidence of potential mild but significant deficits in terms of social novelty recognition as well as repetitive behavior in the BTBR/R mouse subline, although further information on detailed behavioral phenotypes of these mice is needed for a comprehensive understanding in this context.

In addition, it is notable that BTBR/J mice have severely reduced hippocampal commissure (HC) and absent corpus callosum (Wahlsten et al., 2003; Dodero et al., 2013; Ellegood et al., 2013; CC). Since in human juveniles and adults with ASD are often reported to have reduced CC volume (Dougherty et al., 2016; Chen et al., 2017; Temur et al., 2019), BTBR/J mice are useful for genetic, anatomical, and behavioral research on the origin and role of the CC and HC. In contrast, the appearances of the CC and HC of BTBR/R mice were normal, although their quantitative examination is required for evaluation of any morphological abnormality. In order to elucidate this striking morphological difference between two BTBR/R and BTBR/J sublines, we researched whether the genes involved in the CC formation (Suárez et al., 2014a) were included within our DEGs. *Wnt8b*, which is one of genes involved in the development of the CC and axonal connections between the left and right sides of the brain (Suárez et al., 2014b), was downregulated in BTBR/R mice brains. FGF signaling is required for the formation of the CC (Smith et al., 2006). Interestingly, *Fgfr1op2*, which encodes the FGFR1 oncogene partner 2 that regulates FGFR1 kinase activity, was commonly downregulated in BTBR/R mice (in the cerebral cortex and striatum, this study) and BTBR/J (in the cortex and hippocampus, Daimon et al., 2015; and in the cerebellum, Shpyleva et al., 2014). It is yet unclear what genetic alteration(s) generate this drastic morphological change between two sublines and whether the presence and absence of the CC and HC largely impact on their brain function and behavior such as social interaction and repetitive behavior. In other words, BTBR/J mice are a model for ASD subjects completely lacking the CC and HC, while BTBR/R mice may be a model for ASD subjects having intact CC and HC or ones with subtle changes in morphology or neurophysiology. In this study, we found the important similarities and difference between these two BTBR sublines. Further study is needed to clarify these issues.

Our behavioral data indicate potential mild deficits in terms of social novelty recognition and repetitive behavior in BTBR/R mice compared to B6 mice, which is, however, in stark contrast to BTBR/J mice, which are reported to have more severe impairments with regard to autism-related behavior. This also suggests that BTBR/R mice have an autistic-like tendency or susceptibility to autism that may become prominent in BTBR/J mice. Clarifying the differences between the two sublines at the transcriptome level can contribute significantly to understanding the genetics of autism susceptibility.

### Transcriptomic Features of Neurotransmitter Systems

Several studies have addressed the involvement of the excitation/inhibition balance in ASD pathologies, especially in the context of dysregulation of glutamatergic and GABAergic neurotransmission systems in ASD patients and models of ASD (Yizhar et al., 2011; Baudouin et al., 2012; Nelson and Valakh, 2015; Horder et al., 2018; Marotta et al., 2020). In previous pharmacological studies, treatment with the mGluR5 antagonist methyl-6-phenylethynyl-pyridine, AMPA receptor positive allosteric modulators, and the GABA-A agonist gaboxadol rescued social deficits or repetitive behavior in BTBR/J mice (Silverman et al., 2012, 2015; Rhine et al., 2019). In our analysis, we highlighted transcriptomic alterations in glutamatergic and GABAergic signaling pathway genes (Figure 7B), and the corresponding genes were found to be altered not only in BTBR/R but also BTBR/J mice in several instances (Supplementary Table 14). Among the glutamatergic signaling pathway genes, the glutaminase (*Gls*), phospholipase A2 group IVB (*Pla2g4b*), and phospholipase A2 group IVE (*Pla2g4e*) genes were also altered in the hippocampus (Provenzano et al., 2016), dorsal striatum (Oron et al., 2019), and cerebellum (Shpyleva et al., 2014). Of the GABAergic signaling pathway genes, the GABA receptor subunit gamma-2 (*Gabrg2*) and GABA-A receptor subunit alpha2 (*Gabra2*) genes were also altered in the hippocampus (Provenzano et al., 2016), cortex (Daimon et al., 2015), and cerebellum (Shpyleva et al., 2014). In addition, downregulation of *D*-aspartate oxidase (*Ddo*), which is responsible for the degradation of the endogenous NMDA receptor agonist *D*-aspartate, was reported in studies on the hippocampus (Provenzano et al., 2016) and the whole brain (Nuzzo et al., 2020). We also identified some DEGs related to the neurotransmitter signaling pathways for acetylcholine, dopamine, and serotonin, which have also been reported to be involved in ASD (Marotta et al., 2020)—e.g., upregulation of two muscarinic acetylcholine receptors (*Chrm2* and *Chrm3*) and downregulation of the vesicular monoamine transporter 1 (VMAT1, *Slc18a1*), which is predominantly a peripheral VMAT type for neuroendocrine cells. Taken together, there are some difference in the transcriptomic profiles of neurotransmitter signaling pathways, particularly in the glutamatergic and GABAergic signaling pathways that may influence the excitation/inhibition balance, between BTBR/R and B6 mice. The co-expression patterns of these identified DEGs may explain possible differences in corticostriatal neurotransmission systems between BTBR/R and B6 mice, although further studies in this context are warranted.

## Conclusions

In conclusion, we characterized the transcriptomic features of the cerebral cortex and striatum of BTBR/R mice in comparison with B6 mice, using microarray, qRT-PCR, and ISH analyses together with comprehensive bioinformatics approaches. We identified DEGs (upregulated and downregulated) and co-expression as well as interaction RNA networks in BTBR/R mice brains. In addition, the BTBR/R mice data were comprehensively compared to those reported in the previous studies of subjects with ASD as well as ASD animal models, including BTBR/J mice. Our results allow us to propose potentially important genes and ncRNAs that may be associated with brain function and behaviors characteristic to BTBR/R mice that are indicative of an autistic-like phenotype. To contribute further to the understanding of ASD genetics and biology, further studies regarding detailed cellular expression patterns as well as functional aspects of the DEGs in BTBR/R mice brain are required, considering the differences and/or similarities with socially impaired BTBR/J mice and highly social B6 mice.

## Data Availability Statement

The datasets generated for this study are available in the NCBI GEO repository under accession numbers GSE156646 and GSM4735682-4735689.

## Ethics Statement

The animal study was reviewed and approved by Animal Experimentation Committee at The University of Tokyo and Tokyo University of Science.

## Author Contributions

SM designed and performed the experiments, analyzed the data, and wrote the paper. J-nH performed the experiments and wrote the paper. CI performed the experiments and provided funding. HI provided funding. YS and TF conceived the study, provided funding, and wrote the paper. All authors contributed to the article and approved the submitted version.

## Conflict of Interest

The authors declare that the research was conducted in the absence of any commercial or financial relationships that could be construed as a potential conflict of interest.
