# Supplementary material for: Comprehensive Profiling of Gene Expression in the Cerebral Cortex and Striatum of BTBRTF/ArtRbrc Mice Compared to C57BL/6J Mice
Source: Front Cell Neurosci. 2020 Dec 10;14:595607. doi: 10.3389/fncel.2020.595607 (PMC7758463; doi:10.3389/fncel.2020.595607)
Supplement: Supplementary file 15 [file Data_Sheet_1.PDF]

Supplementary Materials

Comprehensive profiling of gene expression in the cerebral cortex and striatum of BTBRTF/ArtRbrc mice compared to C57BL/6J mice

Shota Mizuno[1], Jun-na Hirota[1], Chiaki Ishii[1], Hirohide Iwasaki[2], Yoshitake Sano[1], Teiichi Furuichi[1*]

[1]Department of Applied Biological Science, Faculty of Science and Technology, Tokyo University of Science, 2641 Yamazaki, Noda, Chiba 278-8510, Japan

[2]Department of Anatomy, Gunma University Graduate School of Medicine, 3-39-22 Showa-machi, Maebashi, Gunma 371-8511, Japan

*** Correspondence:**

Teiichi Furuichi

tfuruichi@rs.tus.ac.jp

Fig. S1

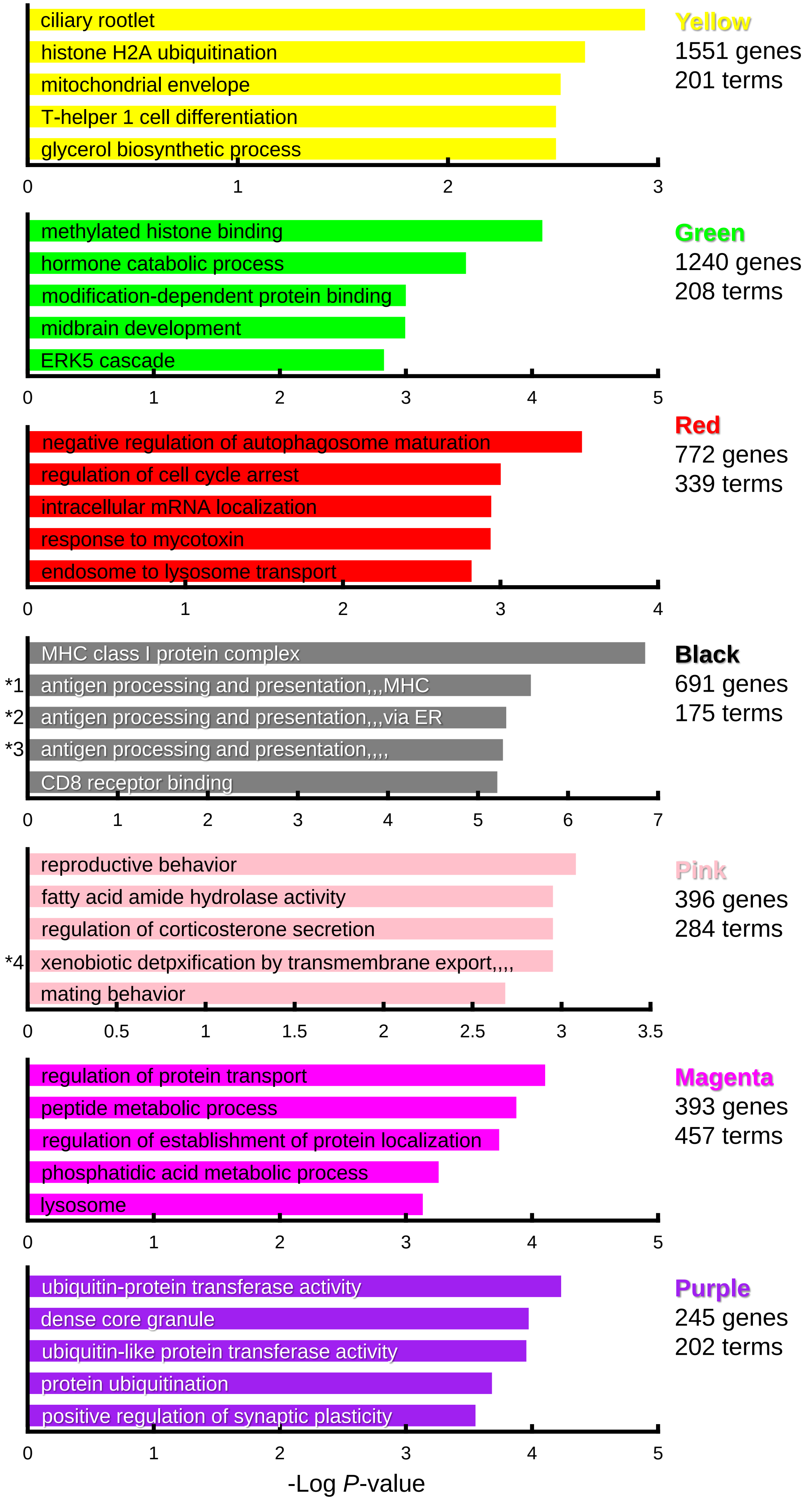
ciliary rootlet
histone H2A ubiquitination
mitochondrial envelope
T-helper 1 cell differentiation
glycerol biosynthetic process
0 1 2 3
Yellow
1551 genes
201 terms
methylated histone binding
hormone catabolic process
modification-dependent protein binding
midbrain development
ERK5 cascade
0 1 2 3 4 5
Green
1240 genes
208 terms
negative regulation of autophagosome maturation
regulation of cell cycle arrest
intracellular mRNA localization
response to mycotoxin
endosome to lysosome transport
0 1 2 3 4
Red
772 genes
339 terms
MHC class I protein complex
*1 antigen processing and presentation,,,MHC
*2 antigen processing and presentation,,,via ER
*3 antigen processing and presentation,,,,
CD8 receptor binding
0 1 2 3 4 5 6 7
Black
691 genes
175 terms
reproductive behavior
fatty acid amide hydrolase activity
regulation of corticosterone secretion
*4 xenobiotic detpxification by transmembrane export,,,,
mating behavior
0 0.5 1 1.5 2 2.5 3 3.5
Pink
396 genes
284 terms
regulation of protein transport
peptide metabolic process
regulation of establishment of protein localization
phosphatidic acid metabolic process
lysosome
0 1 2 3 4 5
Magenta
393 genes
457 terms
ubiquitin-protein transferase activity
dense core granule
ubiquitin-like protein transferase activity
protein ubiquitination
positive regulation of synaptic plasticity
0 1 2 3 4 5
Purple
245 genes
202 terms
-Log P-value

Supplementary Figure 1. Co-expression modules analyzed by weighted correlation network analysis (WGCNA).

The top three modules obtained by WGCNA analysis of differentially expressed genes are shown in Figure 6. The remaining six modules and annotated pathways, “Yellow module”, “Green module”, “Red module”, “Black module, “Pink module”, “Magenta module” and “Purple module”, are indicated. Full name of gene ontology categories for *1–4: 1, antigen processing and presentation of endogenous peptide antigen via MHC class I; 2, antigen processing and presentation of endogenous peptide antigen via MHC class I via endoplasmic reticulum pathway, TAP-independent; 3 antigen processing and presentation of endogenous antigen; 4, xenobiotic detoxification by transmembrane export across the plasma membrane.

Fig. S2

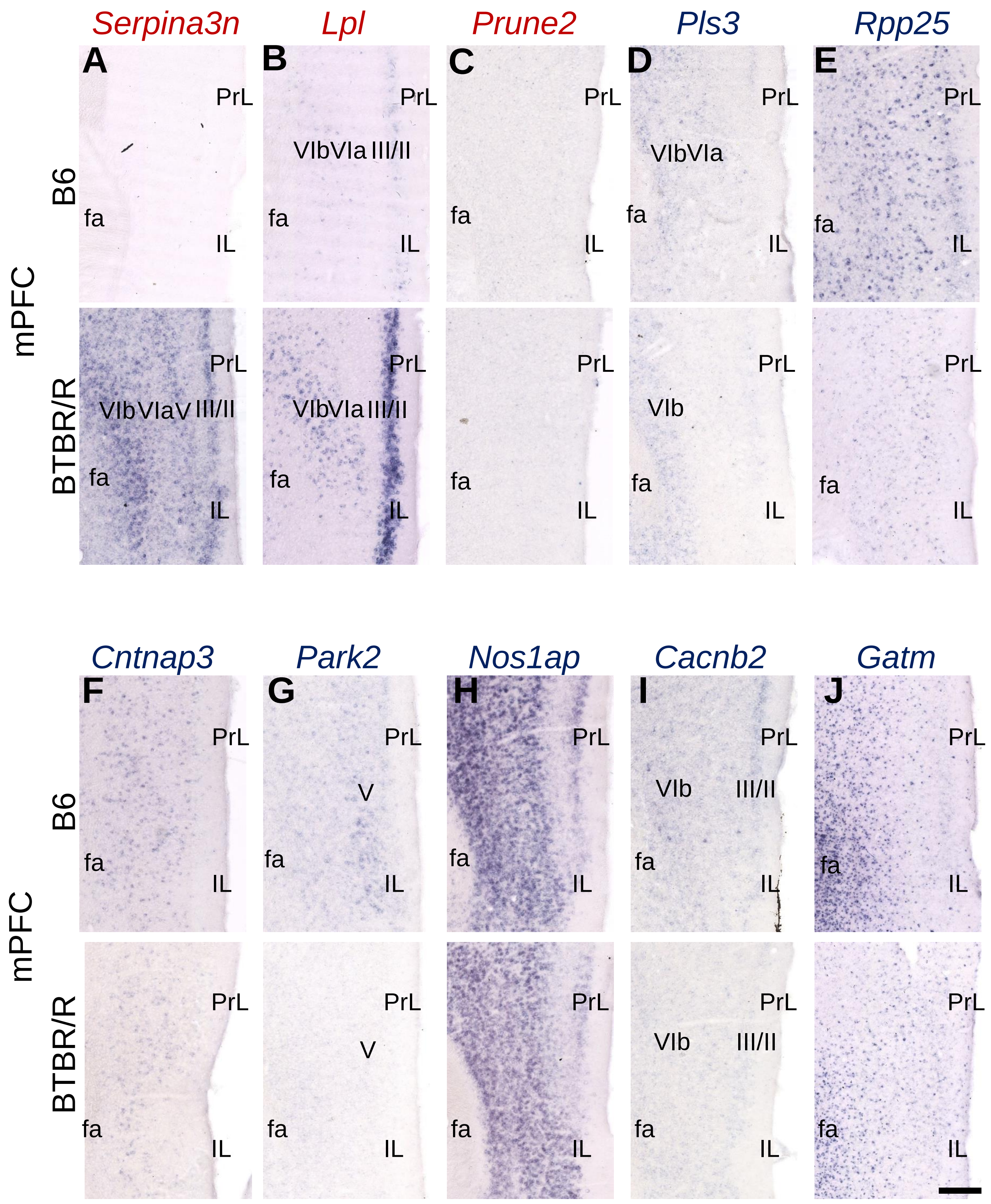

Supplementary Figure S2. Expression pattern of differentially-expressed genes in the medial prefrontal cortex.

In situ hybridization images show the gene expression of *Serpina3n* (A), *Lpl* (B), *Prune2* (C), *Pls3* (D), *Rpp25* (E), *Cntnap3* (F), *Park2* (G), *Nos1ap* (H), *Cacnb2* (I), and *Gatm* (J). Upper and under column shows B6 mice and BTBR/R mice, respectively. Scale bars show 250 μm. I-VIb, cerebral cortical layer I, II/III, V, VIa and VIb; fa, corpus callosum, anterior forceps; IL, infralimbic cortex; PrL, prelimbic cortex. The red and blue letters indicate upregulated and downregulated genes, respectively.

Fig. S3

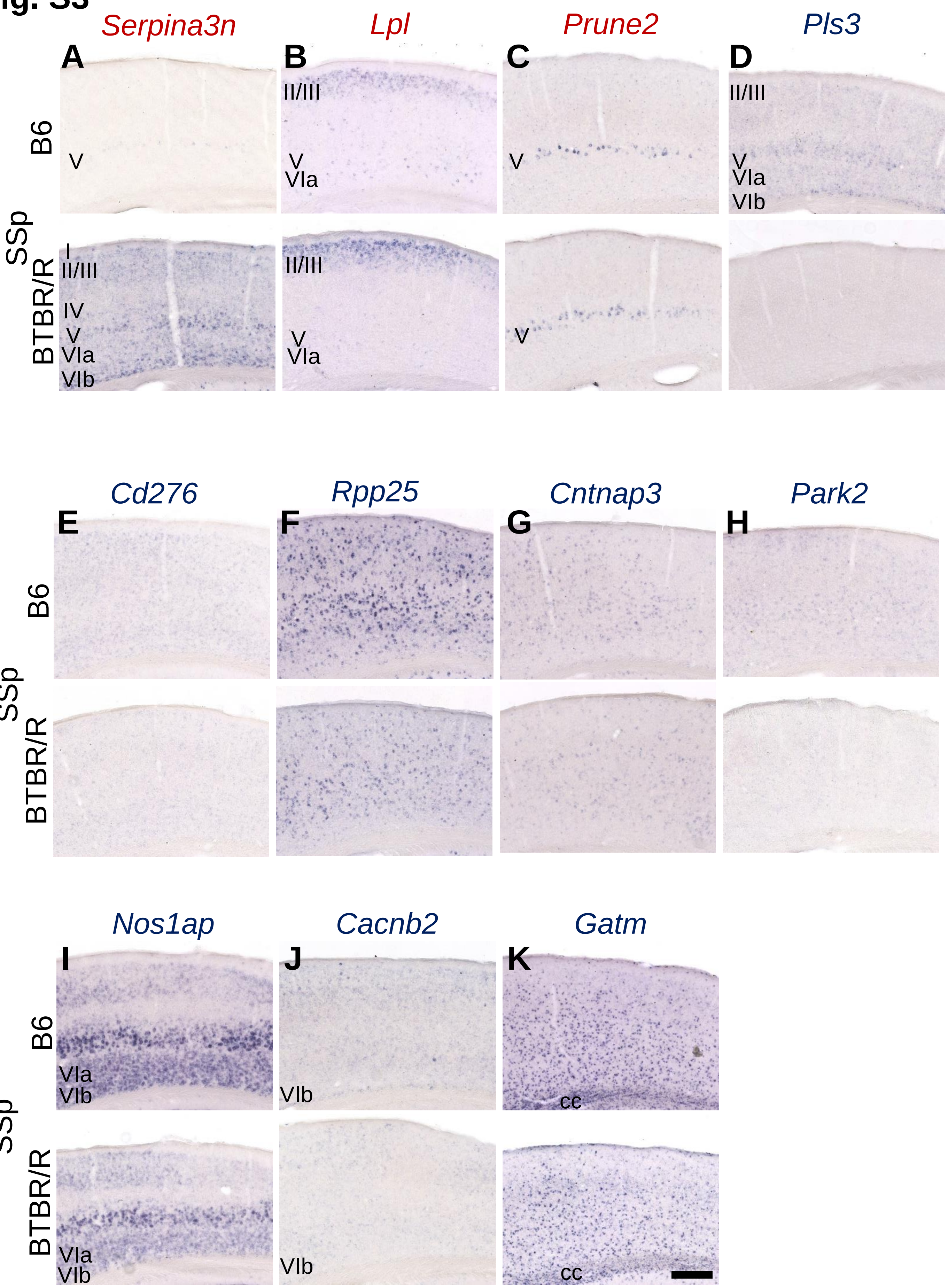

Serpina3n
Lpl
Prune2
Pls3
A
B
C
D
B6
SSp
BTBR/R
I
II/III
IV
V
VIa
VIb
Cd276
Rpp25
Cntnap3
Park2
E
F
G
H
Nos1ap
Cacnb2
Gatm
I
J
K
cc

Supplementary Figure S3. Expression pattern of differentially-expressed genes in the primary somatosensory cortex.

In situ hybridization images show the gene expression of *Serpina3n* (A), *Lpl* (B), *Prune2* (C), *Pls3* (D), *Cd276* (E), *Rpp25* (F), *Cntnap3* (G), *Park2* (H), *Nos1ap* (I), *Cacnb2* (J), and *Gatm* (K). Upper and lower column shows B6 mice and BTBR/R mice, respectively. Scale bars show 250 μm. I-VIb, cerebral cortical layer I, II/III, IV, V, VIa and VIb; cc, corpus callosum. The red and blue letters indicate upregulated and downregulated genes, respectively.

Fig. S4

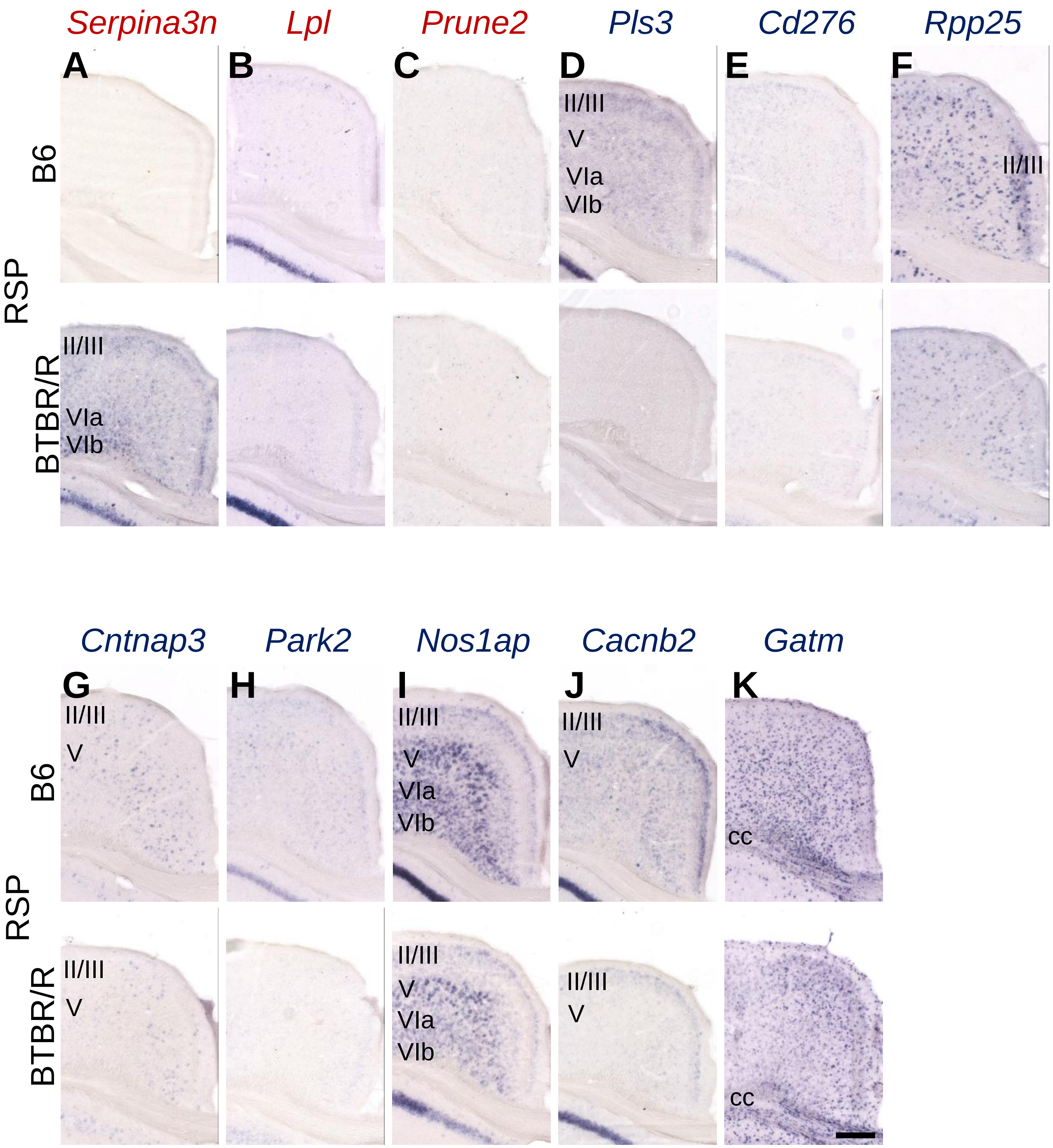

Supplementary Figure S4. Expression pattern of differentially-expressed genes in the retrosplenial cortex.

In situ hybridization images show the gene expression of *Serpina3n* (A), *Lpl* (B), *Prune2* (C), *Pls3* (D), *Cd276* (E), *Rpp25* (F), *Cntnap3* (G), *Park2* (H), *Nos1ap* (I), *Cacnb2* (J), and *Gatm* (K). Upper and lower column shows B6 mice and BTBR/R mice, respectively. Scale bars show 250 μm. I-VIb, cerebral cortical layer I, II/III, V, VIa and VIb; cc, corpus callosum. The red and blue letters indicate upregulated and downregulated genes, respectively.

Fig. S5

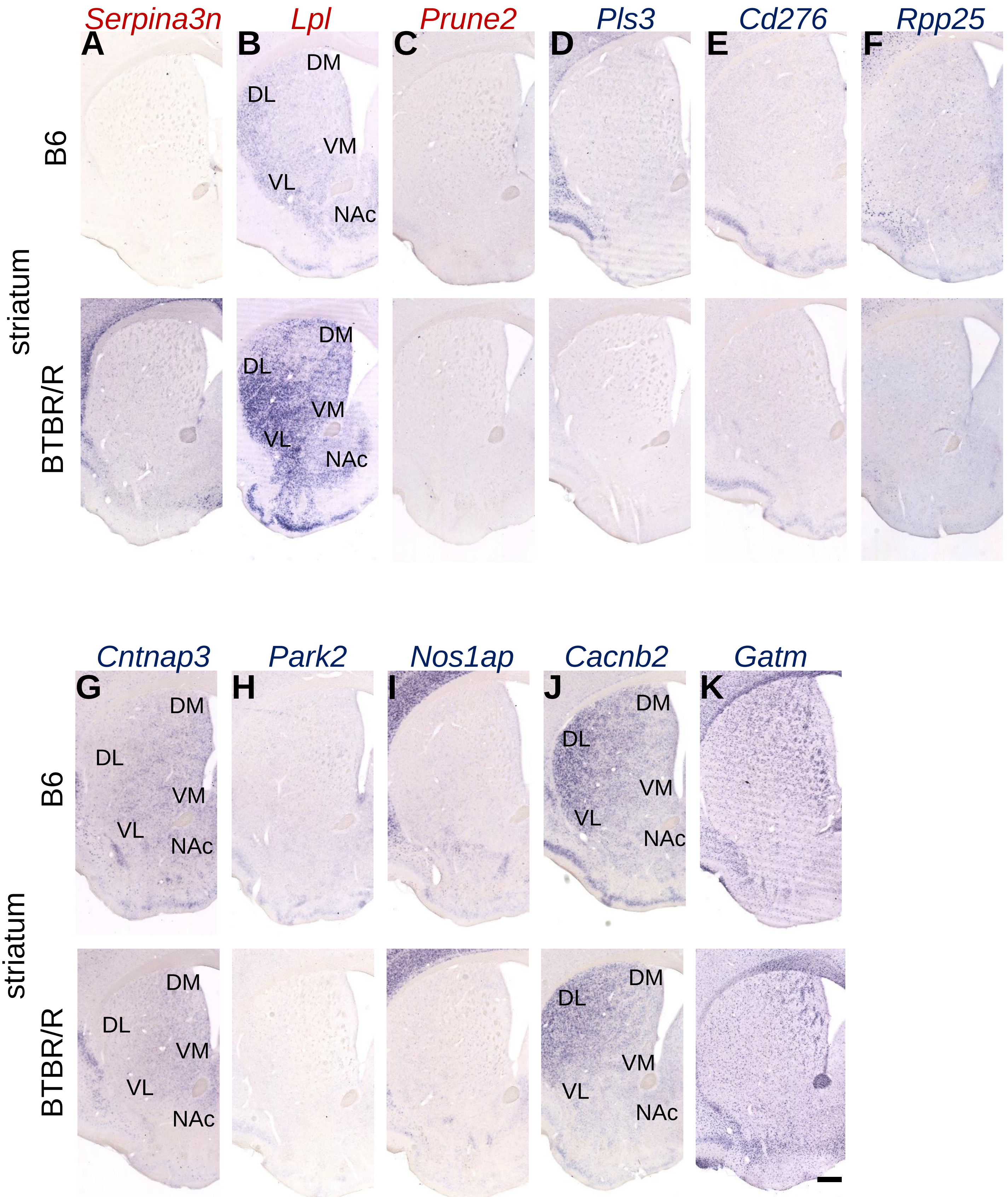

Supplementary Figure S5. Expression pattern of differentially-expressed genes in the striatum. *In situ* hybridization images show the gene expression of *Serpina3n* (A), *Lpl* (B), *Prune2* (C), *Pls3* (D), *Cd276* (E), *Rpp25* (F), *Cntnap3* (G), *Park2* (H), *Nos1ap* (I), *Cacnb2* (J), and *Gatm* (K). Upper and lower column shows B6 mice and BTBR/R mice, respectively. Scale bars show 500 μm DL, dorsolateral striatum; DM, dorsomedial striatum; NAc, nucleus accumbens; VL, ventrolateral striatum; VM, ventromedial striatum. The red and blue letters indicate upregulated and downregulated genes, respectively.

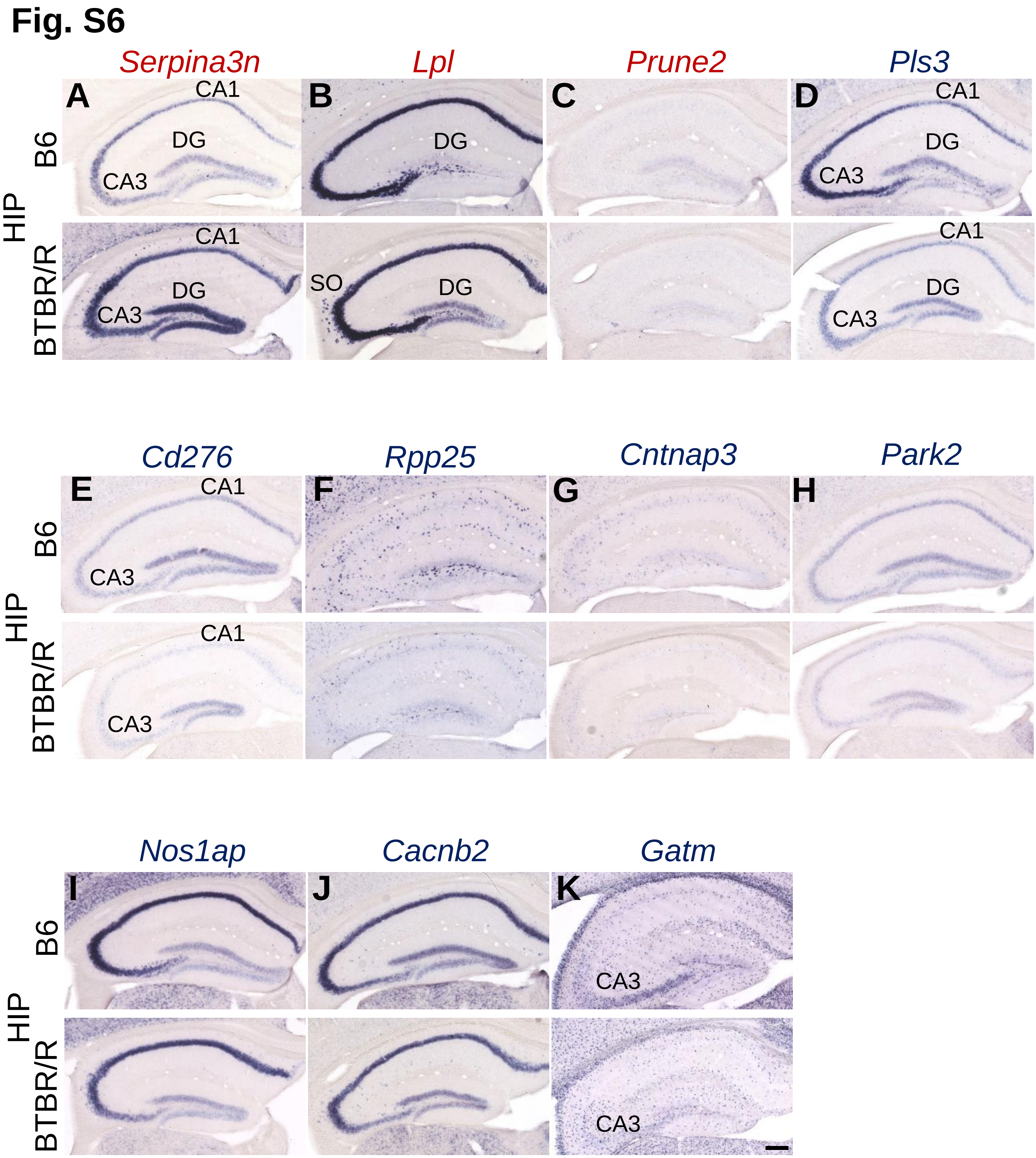

Supplementary Figure S6. Expression pattern of differentially-expressed genes in the hippocampus.

In situ hybridization images show the gene expression of *Serpina3n* (A), *Lpl* (B), *Prune2* (C), *Pls3* (D), *Cd276* (E), *Rpp25* (F), *Cntnap3* (G), *Park2* (H), *Nos1ap* (I), *Cacnb2* (J), and *Gatm* (K). Upper and lower column shows B6 mice and BTBR/R mice, respectively. Scale bars show 250 µm. CA1, pyramidal layer of the cornu ammonis 1; CA3, pyramidal layer of the cornu ammonis 3; DG, dentate gyrus; SO, Stratum oriens. The red and blue letters indicate upregulated and downregulated genes, respectively.

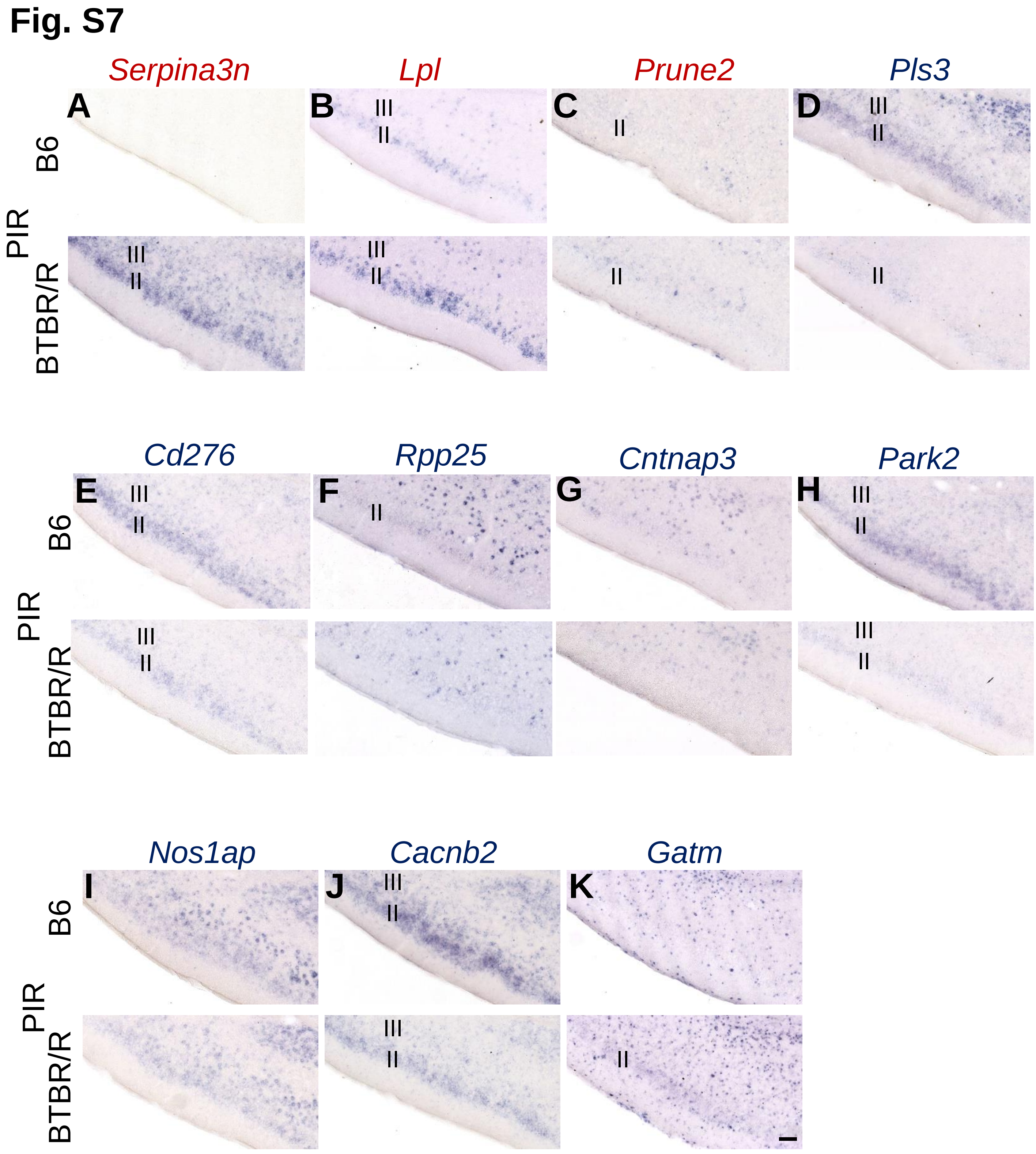

Supplementary Figure S7. Expression pattern of differentially-expressed genes in the piriform cortex.

In situ hybridization images show the gene expression of *Serpina3n* (A), *Lpl* (B), *Prune2* (C), *Pls3* (D), *Cd276* (E), *Rpp25* (F), *Cntnap3* (G), *Park2* (H), *Nos1ap* (I), *Cacnb2* (J), and *Gatm* (K). Upper and lower column shows B6 mice and BTBR/R mice, respectively. Scale bars show 100 µm. II and III, cerebral cortical layer II and III. The red and blue letters indicate upregulated and downregulated genes, respectively.

Fig. S8

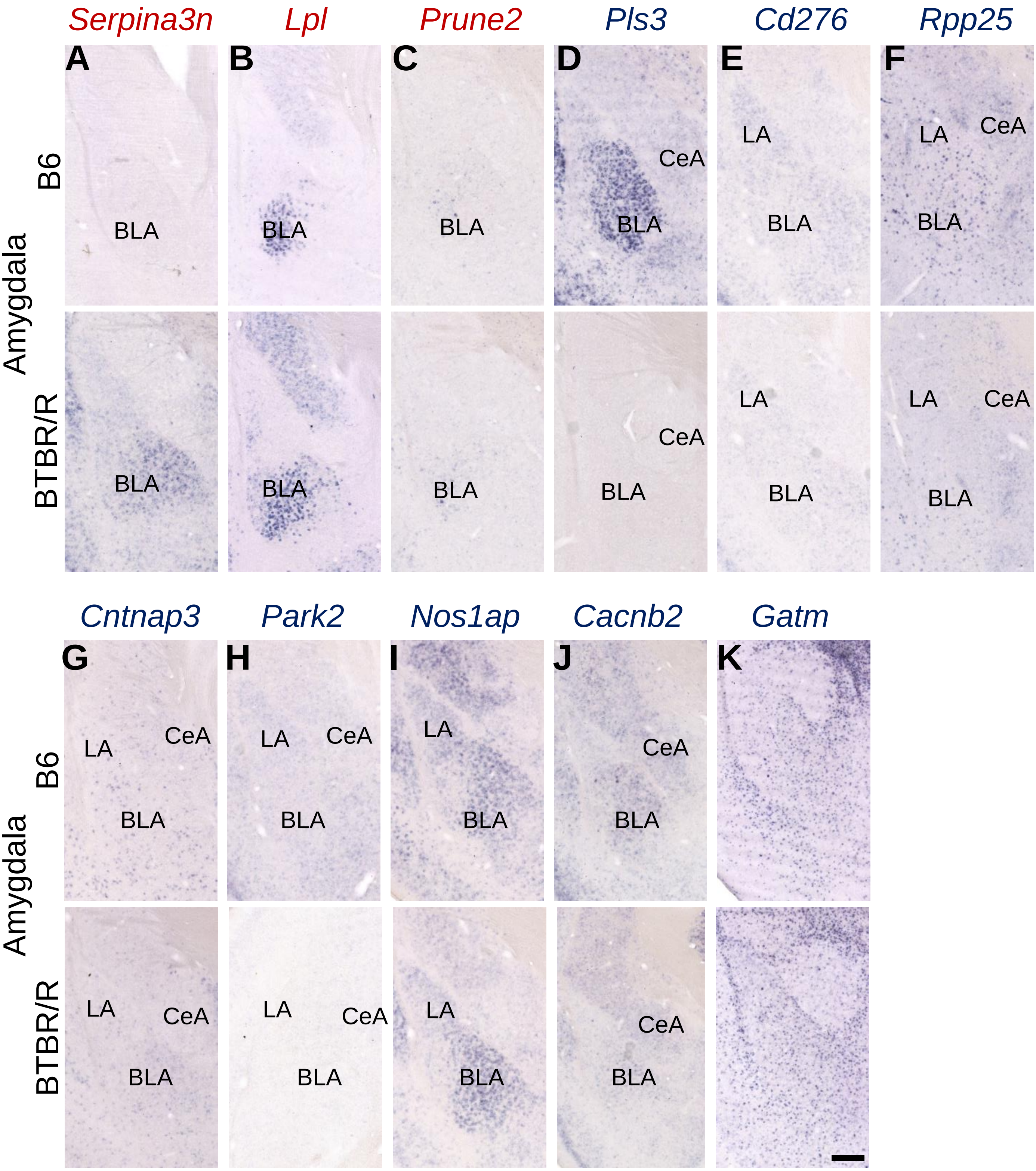

Supplementary Figure S8. Expression pattern of differentially-expressed genes in the amygdala.

In situ hybridization images show the gene expression of *Serpina3n* (A), *Lpl* (B), *Prune2* (C), *Pls3* (D), *Cd276* (E), *Rpp25* (F), *Cntnap3* (G), *Park2* (H), *Nos1ap* (I), *Cacnb2* (J), and *Gatm* (K). Upper and lower column shows B6 mice and BTBR/R mice, respectively. Scale bars show 250 μm. BLA, basolateral amygdala; CeA, central amygdala; LA, lateral amygdala. The red and blue letters indicate upregulated or downregulated genes, respectively.

Fig. S9

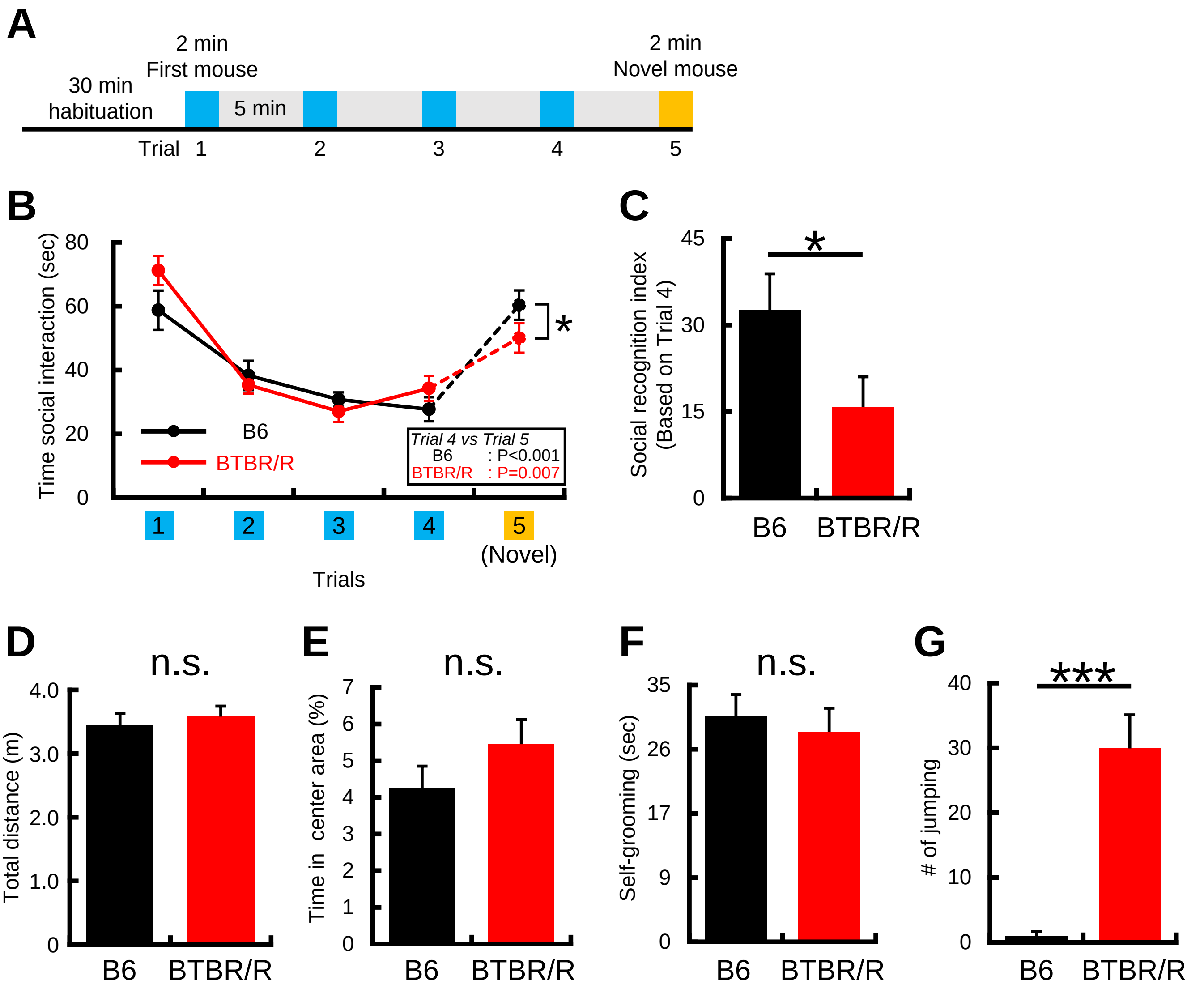

Supplementary Figure S9. Behavioral analysis of BTBR/R and B6 mice.

(A-C) Five-trial social habituation/recognition task. (A) Task schedule. Blue and orange denote the first and the novel social stimulus, respectively. Mean social interaction time (B) and social recognition index (C). BTBR/R mice had a lower social recognition index. (D-G) 10 minutes open field test. Mean distance (D), time in center area (E), time spent self-grooming (F) and jumping (G). Black bars represent B6 mice data, and red bars represent BTBR/R mice data. Social habituation/recognition task: B6, n = 12; BTBR, n = 15. Open field test: B6, n = 21; BTBR n, = 25. Repeated-measures two-way ANOVA followed by Tukey-Kramer post hoc test (B) and unpaired two-sided *t*-test (C-G).* P <0.05, *** P <0.001.

Supplementary Methods

Social recognition task

The social recognition task was performed as described previously, with minor modifications (Ferguson et al., 2000; Hörnberg et al., 2020). Briefly, recognition between elder male test mice (B6 and BTBR/R: 2.7–3.1 and 2.3–2.8 months of age, respectively) and young male stimulus mice (B6 and BTBR/R: 1.2–1.4 and 1.5–1.6 months of age, respectively) was tested with 5 min inter-trial intervals. A new animal home cage was used as the experimental cage. The experimental test mice were acclimated in the cage for 30 min before the start of the test. At the start of the first trial, an unfamiliar stimulus mouse of the same strain was introduced into the cage for 2 min and mice were allowed to freely interact. This was repeated for 4 more consecutive trials with 5 min intervals to allow habituation to the stimulus. On the fifth trial, a novel stimulus mouse was introduced. The interaction time was used to calculate the recognition index as: (interaction trial 5 time) − (interaction trial 4 time).

Open field test

The open field test was performed for adult male BTBR/R (2–6 months of age) and B6 (3-5 months of age) mice using a 33 × 33 × 33 cm white box. Each animal was placed in the center of the arena and freely explored the box under a light intensity of 40 lux for 10 min. Distance traveled and time stayed at the center (the virtually central square, 11 × 11 cm) were analyzed by Ethovison XT (Noldus, Wageningen, Netherlands). The number of rearing, grooming and jumping times were analyzed by hand count.

Statistical analysis

All statistical analyses were performed using Excel 2019 (Microsoft, Redmond, WA, USA). Datasets between two groups were analyzed for significance using unpaired two-tailed Student’s t-tests. In Supplementary Fig. S9B, multiple-group comparisons were performed using repeated-measures two-way ANOVA followed by Tukey-Kramer post hoc test. All data are presented as mean ± SEM. In this study, *P*-values <0.05 were considered significant. Additional information on statistical analysis is described in the corresponding subsections of the Materials and Methods section and the figure legends.
